# Downregulated expression of ARHGAP10 correlates with advanced stage and high Ki-67 index in breast cancer

**DOI:** 10.7717/peerj.7431

**Published:** 2019-08-01

**Authors:** Yujing Li, Beilei Zeng, Yunhai Li, Chong Zhang, Guosheng Ren

**Affiliations:** 1 Chongqing Key Laboratory of Molecular Oncology and Epigenetics, The First Affiliated Hospital of Chongqing Medical University, Chongqing, China; 2 Department of Ultrasound, The First Affiliated Hospital of Chongqing Medical University, Chongqing, China; 3 Department of Endocrine and Breast Surgery, The First Affiliated Hospital of Chongqing Medical University, Chongqing, China

**Keywords:** ARHGAP10, Breast cancer, TCGA, Oncomine, Immunohistochemistry, GEO2R, GSEA

## Abstract

**Background:**

Rho GTPase-activating protein 10 (ARHGAP10), which catalyzes the conversion of active Rho GTPase to the inactive form, is downregulated in some cancers. However, little is known about ARHGAP10 in breast cancer.

**Methods:**

The transcriptional expression level of ARHGAP10 in breast cancer was analyzed with the data downloaded from The Cancer Genome Atlas (TCGA) and Oncomine, then verified by reverse-transcription quantitative polymerase chain reaction (RT-qPCR) in 30 pairs of breast cancer tissues and the corresponding adjacent normal tissues. ARHGAP10 protein expression was examined by immunohistochemistry (IHC) in 190 breast cancer and 30 corresponding adjacent normal breast tissue samples. The associations between ARHGAP10 expression and clinicopathological characteristics of patients were analyzed, and Kaplan–Meier Plotter was used to assess the relationship between ARHGAP10 and relapse-free survival (RFS). Different expression levels of ARHGAP10 in response to chemotherapy agents were determined by GEO2R online tool. The potential biological functions of ARHGAP10 were analyzed by Gene Set Enrichment Analysis (GSEA) using data downloaded from TCGA.

**Results:**

ARHGAP10 mRNA and protein expression was lower in breast cancer tissues than in adjacent normal tissues. Low expression of ARHGAP10 was associated with advanced clinical TNM (cTNM) stage (*p*_*b*_ = 0.001) and high Ki-67 index (*p* = 0.015). Low expression of ARHGAP10 indicated worse RFS (*p* = 0.0015) and a poor response to chemotherapy (*p* = 0.006). GSEA results showed that ARHGAP10 was involved in signaling pathways including protein export, nucleotide excision repair, base excision repair, focal adhesion, JAK-STAT pathway and the actin cytoskeleton.

## Introduction

Breast cancer is the most prevalent malignant disease with highest incidence in females worldwide, accounting for 25% of all cancer cases and 15% of all cancer-related deaths among women according to the updated global cancer statistics (Chen et al., 2016). Treatment decisions for breast cancer largely depend on clinicopathological parameters (tumor size, hormone receptor status, HER2 status, Ki-67 index, pathological grade, lymph node metastasis and distant metastasis). However, the management of breast cancer remains unsatisfactory, and the responses to treatment and patient outcomes vary among individuals partly because of the heterogeneity of tumors (Song et al., 2016). Therefore, the identification of new biomarkers and elucidating the molecular mechanisms underlying breast cancer are critical to determine the effectiveness of breast cancer treatments.

ARHGAP10, also known as GRAF2 or PS-GAP, is located at chromosome 4q31.23 (Katoh & Katoh, 2004). ARHGAP10, a member of the Rho GTPase-activating protein (GAP) family, which includes at least 49 members (Fagerberg et al., 2014), reverses the active GTP-bound form of GTPases into an inactive GDP-bound form (Lancaster et al., 1994). Rho GAP family members have been investigated for their involvement in several cancers including lung cancer (Wang et al., 2014), hepatocellular carcinoma (Gen et al., 2009), prostate cancer (Lazarini et al., 2013), colorectal cancer (Johnstone et al., 2004), Ewing carcinoma (Satterfield et al., 2017) and glioblastoma (Bigarella et al., 2009) among others. The activity of GTPases such as Cdc42 and RhoA is modulated by ARHGAP10 (Shibata et al., 2001). ARHGAP10 contains four domains, namely BAR, PH, Rho GAP, and SH3 (Katoh & Katoh, 2004), which mediate its interactions with proteins such as FAK (Ren et al., 2001), PAK2 (Koeppel et al., 2004) and PKN β (Shibata et al., 2001). These interactions mediate the cell growth, cell shape, motility, exintegrin-initiated signaling events, cell survival, cell death, cell growth and cell apoptosis. Consequently, aberrant expression of ARHGAP10 may relate to disorders in diseases.

ARHGAP10 acts as tumor suppressor in ovarian cancer (Luo et al., 2016), lung cancer (Teng et al., 2017) and gastric cancer (Li et al., 2017). ARHGAP10 expression is modulated by micro-RNA and is involved in signaling cascades such as Wnt pathway, metastasis, cell cycle, replication, base excision repair, mitochondria-dependent apoptosis and autophagic cell death. ARHGAP10 also affects the expression of p53 (Li et al., 2017), β-catenin, MMP-9, MMP-2 (Teng et al., 2017), PCNA, PLK, MCM2, MCM3 and PARP (Luo et al., 2016). An association between single nucleotide polymorphism in the intron of ARHGAP10 and the prognosis of breast cancer was reported, although it did not reach strict statistical significance and the relation could not be replicated in the validation cohort (Azzato et al., 2010). Therefore, ARHGAP10 in breast cancer remains to be investigated in detail.

The present study was intended to investigate the expression pattern and potential biological function of ARHGAP10 in breast cancer. We confirmed that ARHGAP10 was significantly downregulated in breast cancer, and low expression of ARHGAP10 was related to a high Ki-67 index, advanced cTNM stage and low RFS rate. GEO data were analyzed to assess the possible role of ARHGAP10 in the response to chemotherapy. Additionally, we conducted GSEA to identify the possible biological functions and potential signaling pathways related to ARHGAP10 in breast cancer.

## Materials & Methods

### RNA extraction and reverse-transcription quantitative polymerase chain reaction

Paired tumor tissues and adjacent normal tissues used in RT-qPCR were obtained from patients who were diagnosed by Department of Pathology and underwent breast cancer surgery at Department of Endocrine and Breast Surgery, the First Affiliated Hospital of Chongqing Medical University. This study was approved by the Institutional Ethics Committees of the First Affiliated Hospital of Chongqing Medical University (#2017-012). Specimens were collected and stored at −80 °C until utilized. Total RNA was isolated using TRIzol reagent (Invitrogen, Carlsbad, CA, USA). The concentration of total RNA was measured with NanoDrop 2000 spectrophotometer (Thermo Fisher Scientific, Waltham, MA, USA). Reverse transcription was performed using Go-Taq polymerase (Promega, Madison, WI, USA). A SYBR Green PCR Master Mix kit (Invitrogen) for RT-qPCR was used with Applied Biosystems 7500 (Applied Biosystems, Foster City, CA, USA). GAPDH was used as internal control. Relative expression was determined by 2^(−Δ*t*)^. Forward primer of ARHGAP10 was TGTGGAACCTATGCTGTCAT and reverse primer of ARHGAP10 was GACTTGCTCGTTTGTGGTC. Forward primer of GAPDH was GGAGTCAACGGATTTGGT and reverse primer of GAPDH was GTGATGGGATTTCCATTGAT.

### Immunohistochemistry

Formalin-fixed and paraffin-embedded tissue samples were obtained from the Department of Endocrine and Breast Surgery and the Department of Pathology, First Affiliated Hospital of Chongqing Medical University from 2012 to 2017. Specimens were sliced into 4 μm thick section. None of the patients in this cohort received anti-tumor treatment before surgery. This study was approved by the Institutional Ethics Committees of the First Affiliated Hospital of Chongqing Medical University (#2017-012). The slides were deparaffinized with fresh xylene for 30 min and rehydrated with graded ethanol for 25 min, then washed with PBS. Antigens were retrieved in a microwave oven at 100 °C for 20 min. Slides were blocked with 3% hydrogen peroxide for 12 min after cooling to ambient temperature, followed by washing in PBS, blocking with normal goat serum for 15 min, incubation with primary antibody against ARHGAP10 (55139-1-AP; Proteintech Group, Wuhan, China) at dilution of 1:250 overnight at 4 °C, and rinsing with PBS. Slides were incubated with biotinylated goat anti-rabbit IgG at 37 °C for 15 min, rinsed with PBS, incubated with horseradish-peroxidase labled streptomycin, and then stained with DAB and hematoxylin respectively. Slides were finally immersed in differentiation liquid. All slides were dehydrated with graded ethanol and xylene series, then fixed with neutral resin and imaged under a microscope. The IHC results were scored according to the proportion of positively stained cells and staining intensity. The final score was determined by the product of staining intensity (0: negative; 1: weak; 2: moderate; 3: strong) and the percentage of positive cells (0: <5%; 1: 5%–25%; 2: 26%–50%; 3: 51%–75%; 4: >75%). A final score <8 was considered as low expression, and ≥8 was considered as high expression.

### Cell lines

The human normal breast epithelial cell line MCF10A and the human breast cancer cell lines MCF7, BT-549, MDA-MB-468 and MDA-MB-231 were obtained from American Type Culture Collection. MCF10A were cultured as previously described (Debnath, Muthuswamy & Brugge, 2003), and other breast cancer cell lines were cultured in RPMI-1640 medium (Gibco-BRL, Karlsruhe, Germany), supplemented with 10% fetal bovine serum (Gibco-BRL) in a 5% CO_2_ humidified atmosphere at 37 °C.

### Antibodies and western blotting

Total cell lysates were lysed in lysis buffer (Beyotime) containing PMSF and an inhibitor cocktail on ice. Protein concentration was measured using the BCA protein assay kit (Pierce, Rockford, IL, USA). Aliquots containing 30 µg protein separated by 10% sodium dodecyl sulfate–polyacrylamide gel electrophoresis, then transferred to polyvinylidene difluoride membranes and blocked with 5% non-fat milk for 1 h at room temperature. Membranes were incubated with the corresponding primary antibody (ARHGAP10 at the dilution of 1:2000 and GAPDH at the dilution of 1:5000) at 4 °C overnight, washed with 0.1% Tween20 in TBS, and then incubated with secondary antibody. Primary rabbit anti-ARHGAP10 and anti-GAPDH were purchased from Proteintech Group (55139-1-AP; Wuhan, China) and BIOSS (bs-10900R, China), respectively. The secondary antibody HRP-conjugated goat anti-rabbit IgG was purchased from Abbkine (A21020, China).

### Bioinformatics resource and data

The significance of ARHGAP10 for RFS in breast cancer was plotted via Kaplan–Meier Plotter (http://www.kmplot.com) (Gyorffy et al., 2010). A total of 3955 breast cancer patients were divided into high and low expression groups according to the median expression of ARHGAP10.

Oncomine (http://www.oncomine.org), an online database of the transcript level of certain genes (Rhodes et al., 2004), was used to examine the differences in gene expression between breast cancer tissues and normal mammary tissues. The following search parameters were used: ARHGAP10, Cancer vs. Normal Analysis, Breast Cancer, Clinical Specimen, ordered by under-expression, *p*-value 0.01 and fold change 2.

GEPIA (http://gepia.cancer-pku.cn/), an online website for RNA sequencing expression data of various tumor and normal samples from TCGA and the GTEx projects (Tang et al., 2017), was used for the analysis.

CpG islands were determined by online tool EMBOSS Cpgplot. CpG islands were defined as sequence ranges where the Obs/Exp value was greater than 0.6 and the GC content was greater than 50% within a length more than 200bp (Gardiner-Garden & Frommer, 1987).

Breast cancer data of 1,097 breast cancer cases and 113 normal cases from TCGA were downloaded from https://cancergenome.nih.gov/.

GSEA was performed using the c2.cp.kegg.v6.2symbols.gmt gene set with GSEA software. A total of 1,097 breast cancer samples were divided into two groups according to the median expression of ARHGAP10, namely the ARHGAP10-low group and ARHGAP10-high group. A nominal *p*-value <0.05 and false discovery rate (FDR) >0.25 were regarded as statistically enriched for a certain gene set.

### Statistical analysis

Statistical analyses were performed with SPSS 23.0 (Chicago, IL, USA) and GraphPad Prism 7 software (San Diego, CA, USA). Two tailed Student’s *t*-test was used to examine the statistical relationship between two cohorts. The chi-square test, Bonferroni correction and Fisher’s exact test were used to examine correlations between clinicopathological parameters and the expression level of ARHGAP10. A *p* < 0.05 was considered statistically significant except in Bonferroni correction. The *p* value using Bonferroni correction was represented as *p*_b_.

## Results

### ARHGAP10 is downregulated at the transcriptional level in breast cancer tissues

**Figure 1:**
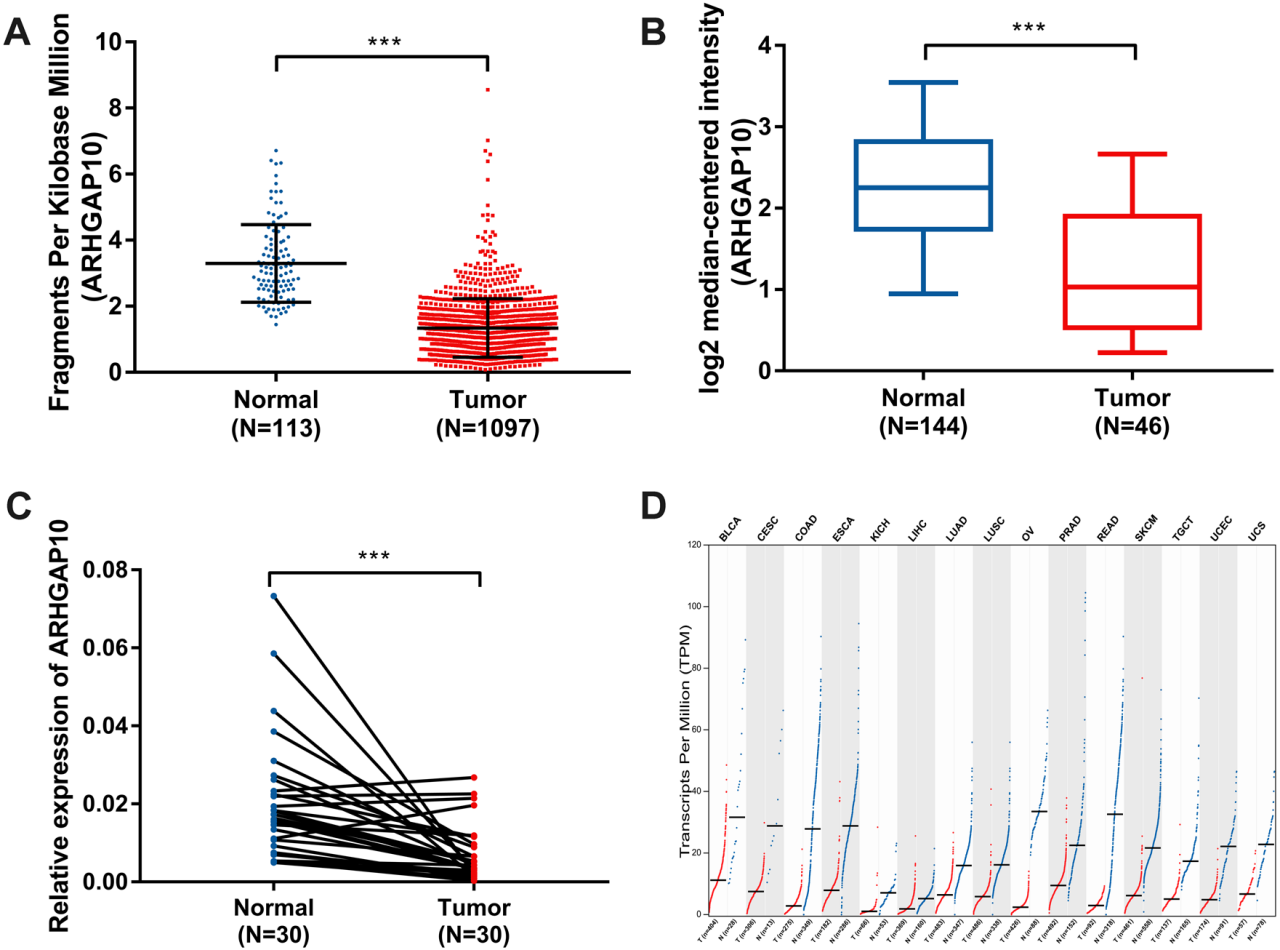
Decreased expression of ARHGAP10 in breast cancer and other types of cancer tissues at transcriptional level. (A) The expression of ARHGAP10 in breast cancer tissues (*n* = 1, 097) and normal breast tissues (*n* = 113) according to TCGA, *p* < 0.001. (B) The expression of ARHGAP10 in breast cancer tissues (*n* = 144) and normal breast tissues (*n* = 46) with the use of data from Oncomine, *p* < 0.001. (C) The expression of ARHGAP10 in 30 paired primary breast cancer tissues and corresponding normal adjacent tissues detected by RT-qPCR assay and examined by paired *t*-test, *p* < 0.001. (D) The downregulated expression of ARHGAP10 in other types of cancer according to GEPIA. BLCA, bladder urothelial carcinoma; CESC, cervical squamous cell carcinoma and endocervical adenocarcinoma; COAD, colon adenocarcinoma; ESCA, esophageal carcinoma; KICH, kidney chromophobe; LIHC, liver hepatocellular carcinoma; LUAD, lung adenocarcinoma; LUSC, lung squamous cell carcinoma; PRAD, prostate adenocarcinoma; READ, rectum adenocarcinoma; SKCM, skin cutaneous melanoma; TGCT, testicular germ cell tumors; UCEC, uterine corpus endometrial carcinoma; UCS, uterine carcinosarcoma.

The expression of ARHGAP10 was analyzed using data downloaded from TCGA and Curtis Breast in Oncomine (Curtis et al., 2012). The results showed that ARHGAP10 was downregulated according to TCGA (*p* < 0.001) (Fig. 1A) and Curtis Breast of Oncomine (*p* < 0.001) (Fig. 1B).

RT-qPCR analysis of 30 paired breast cancer and corresponding adjacent normal tissues was performed to confirm the expression pattern of ARHGAP10 in breast cancer. The result showed that ARHGAP10 was decreased significantly at transcriptional level when compared with paired normal adjacent tissues (*p* < 0.001) (Fig. 1C).

Intriguingly, ARHGAP10 was not only downregulated in breast cancer, but was also reduced in other cancers including bladder urothelial carcinoma, cervical squamous cell carcinoma and endocervical adenocarcinoma, colon adenocarcinoma, esophageal carcinoma, kidney chromophobe, liver hepatocellular carcinoma, lung adenocarcinoma, lung squamous cell carcinoma, prostate adenocarcinoma, rectum adenocarcinoma, testicular germ cell tumor, uterine corpus endometrial carcinoma and uterine carcinosarcoma according to GEPIA (Tang et al., 2017) (Fig. 1D).

### ARHGAP10 protein expression is downregulated in breast cancer tissues

**Figure 2:**
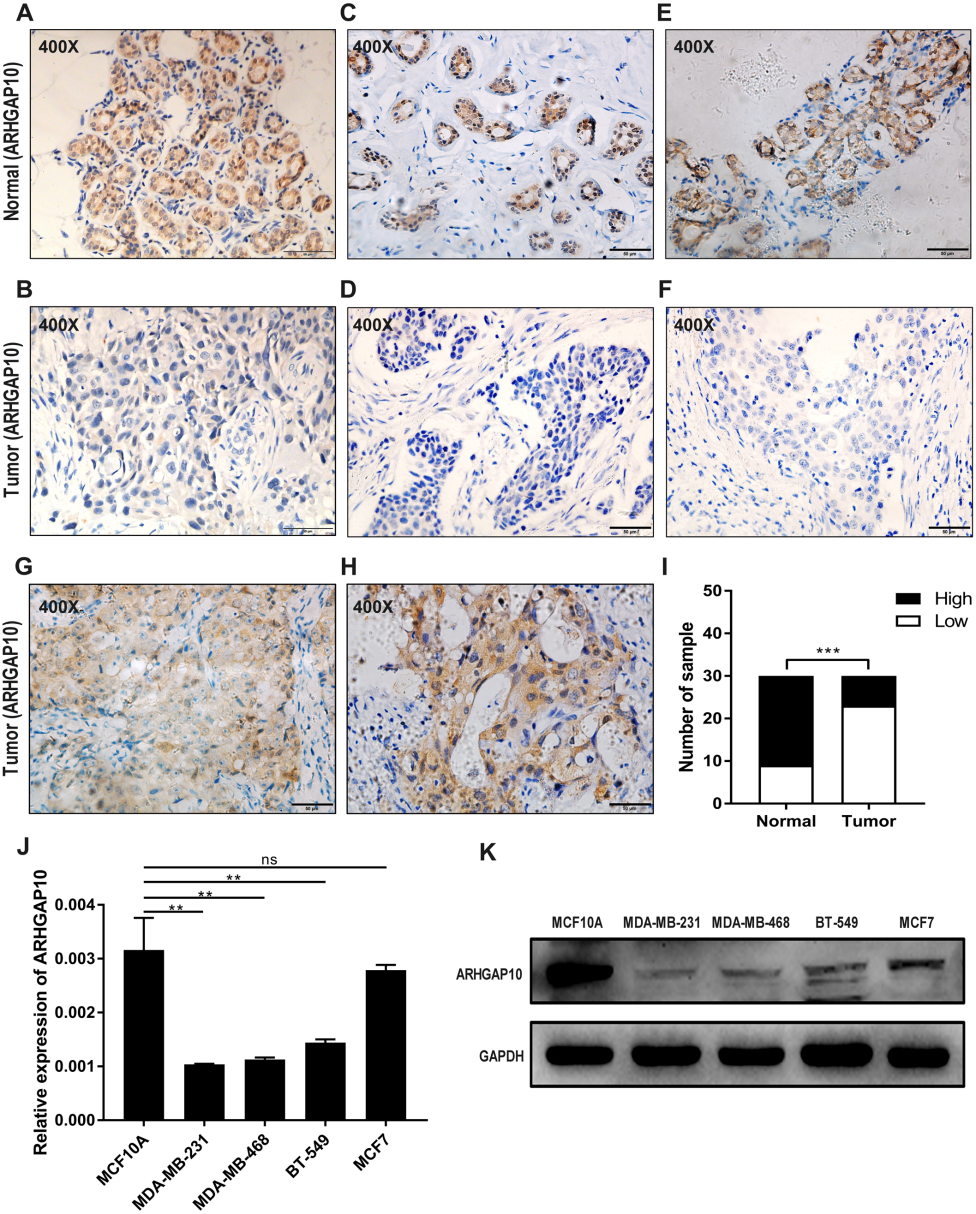
Reduced protein expression of ARHGAP10 in breast cancer tissues and expression pattern of ARHGAP10 in breast cell lines. (A–B, C–D and E–F are three pairs of representative IHC images of high-ARHGAP10 expression in normal adjacent breast tissues and low-ARHGAP10 expression in corresponding breast cancer tissues. Scale bars, 50 μm. (G–H) Representative IHC images of high-ARHGAP10 expression breast cancer tissues. Scale bars, 50 μm. (I) Distributions of different expression levels of ARHGAP10 in 30 paired tumor and normal adjacent tissues. The statistical significance was examined by Chi-square test, *p* < 0.001. (J) Expression of ARHGAP10 in MCF10A and breast cancer cell lines MDAMB-231, MDA-MB-468, BT-549 and MCF7 were evaluated by RT-qPCR and analyzed by Student’s test, ** *p* < 0.01. (K) Expression of ARHGAP10 in MCF10A and breast cancer cell lines MDAMB-231, MDA-MB-468, BT-549 and MCF7 examined by western blotting.

The protein expression of ARHGAP10 was analyzed in 30 paired tumor tissues and corresponding adjacent normal tissues by IHC. ARHGAP10 was located at both the cytoplasm and nucleus (Figs. 2A–2H). The IHC staining results, as determined by the product of the proportion of positive staining cells and staining intensity, showed that low expression of ARHGAP10 was more frequent in breast cancer tissues than in non-cancerous tissues (*p* < 0.001) (Fig. 2I).

The expression of ARHGAP10 in breast cell lines was evaluated by RT-qPCR and western blotting. The results demonstrated that ARHGAP10 was significantly lower in MDA-MB-231, MDA-MB-468 and BT-549 when compared with MCF10A, and ARHGAP10 showed relatively low-expression trend in MCF7, though it was of no statistical significance at mRNA level (Figs. 2J and 2K). The expression trend of ARHGAP10 in breast cancer cell lines was roughly consistent with that in the clinical tissue samples.

**Table 1:** Correlation between the clinicopathological parameters and the expression of ARHGAP10 in 190 breast cancer cases.

**Clinicopathologicalcharacteristics**	**Number**	**ARHGAP10 expression**		***p*value**
	(*n* = 190)	Low	High	
**Age**				
<40	29	23	6	
≥40	161	112	49	0.287
**Size**				
≤20 mm	57	38	19	
>20 mm ≤50 mm	107	81	26	
≥50 mm	0	0	0	
unknown	26	16	10	0.217
**ER Status**				
Negative	87	59	28	
Positive	103	76	27	0.366
**PR Status**				
Negative	98	66	32	
Positive	92	69	23	0.245
**HER2**				
Negative	108	80	28	
Positive	55	35	20	
Unknown	27	20	7	0.167
**Ki-67**				
≤20%	81	50	31	
>20%	109	85	24	0.015
**Pathological grade**				
1	3	2	1	
2	135	94	41	
3	40	32	8	
unknown	12	7	5	0.379
**Lymph node metastasis**				
Negative	111	75	36	
Positive	79	60	19	0.209
**cTNM**				
I	63	35	28	
II	122	96	26	
III	5	4	1	0.005

### ARHGAP10 expression correlates with Ki-67 index and cTNM stage

To gain insight into the significance of ARHGAP10 in breast cancer, a total of 190 breast cancer samples were analyzed. In these samples, 71% (135/190) of tumor tissues showed low expression of ARHGAP10. Previous studies of ARHGAP10 in cancer did not examine the relationship between the expression of ARHGAP10 at protein level and clinicopathological parameters, which are important for patient prognosis and therapy options. We therefore divided patients into different groups by age, tumor size, ER status, PR status, HER-2 status, Ki-67 index (Ki-67 ≤20% was considered as low Ki-67 index, Ki-67 >20% was considered as high Ki-67 index), lymph node metastasis, pathological grade and cTNM stage according to the medical records with stratified expression level of ARHGAP10. ARHGAP10 expression was significantly correlated with Ki-67 index (*p* = 0.015) and clinical TNM stage (*p* = 0.005). Low expression of ARHGAP10 was significantly more frequent in cases with relatively high Ki67 index and advanced clinical stage (*p*_*b*_ = 0.001). However, ARHGAP10 expression was not significantly correlated with age (*p* = 0.287), tumor size (*p* = 0.217), ER status (*p* = 0.366), PR status (*p* = 0.245), HER2 (*p* = 0.167), pathological grade (*p* = 0.379) and lymph node metastasis (*p* = 0.209). Clinicopathological characteristics and expression level of ARHGAP10 of these breast cancer samples were summarized in Table 1.

### Low expression of ARHGAP10 is associated with poor RFS and response to chemotherapy

**Figure 3:**
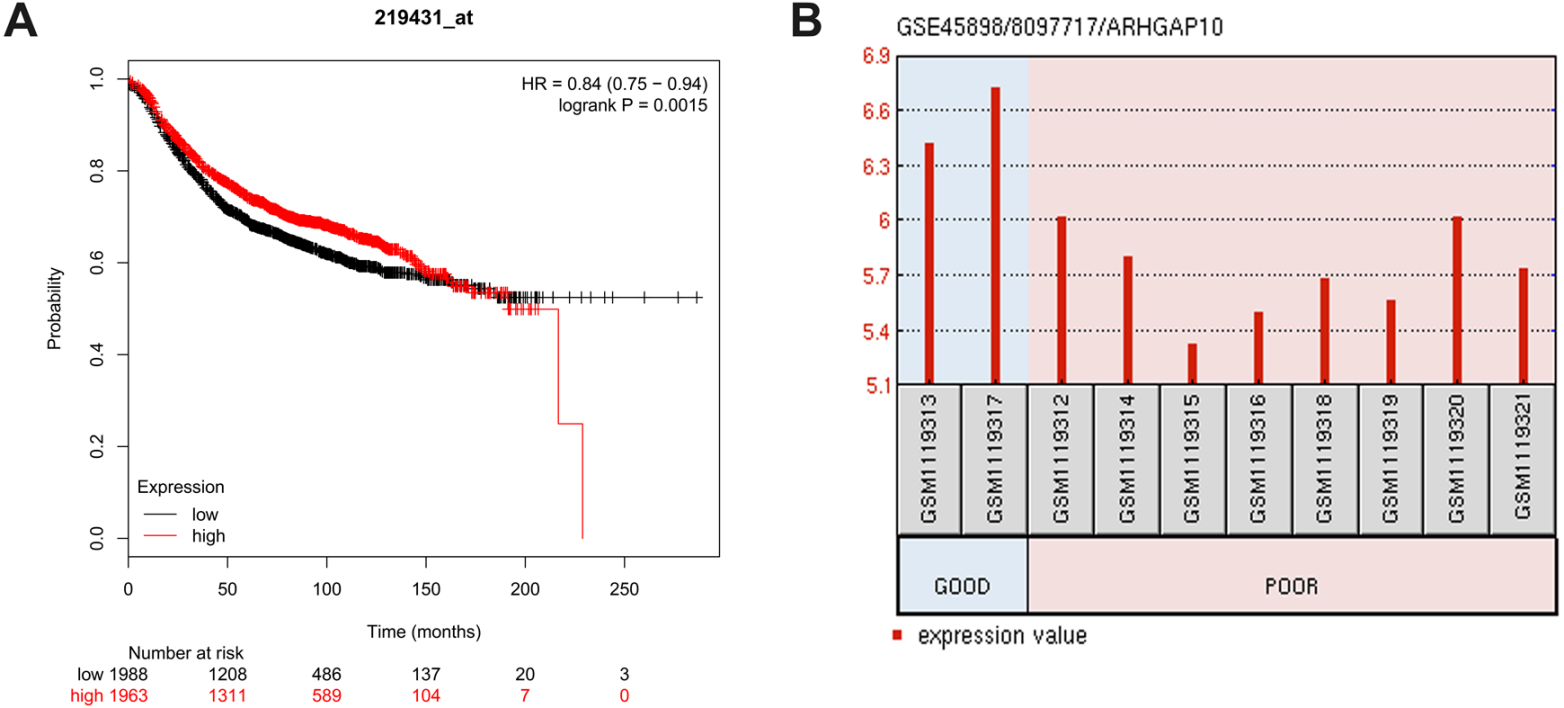
Correlation between the expression level of ARHGAP10 and relapse free survival (RFS) and the responses to the chemotherapeutic agents. (A) The prognostic value of ARHGAP10 about RFS assessed by Kaplan Meier Plotter which contains 3955 cases divided by the median expression of ARHGAP10. HR = 0.84 (0.75–0.94), *p* = 0.0015. (B) The expression of ARHGAP10 in different drug response groups analyzed by GEO2R with GSE45898.

The relationship between ARHGAP10 and RFS was analyzed using Kaplan–Meier Plotter in 3,955 breast cancer cases (Gyorffy et al., 2010). The results showed that low expression of ARHGAP10 was associated with a lower RFS rate after dividing patients according to the median ARHGAP10 expression (*p* = 0.0015) (Fig. 3A). This suggested that ARHGAP10 could be a predictor of breast cancer prognosis.

GSE45898 is a gene expression profile of breast cancer patients with contrary responses to epirubicin, cyclophosphamide and docetaxel chemotherapy (Gruosso et al., 2016). In this dataset, breast cancer patients’ biopsies were analyzed before therapy, and the responses to chemotherapy were classified as Good and Poor. Differentially expressed mRNAs were analyzed using GEO2R, and ARHGAP10 was identified as a differently expressed transcript with statistical significance. ARHGAP10 expression was significantly lower in the Poor group and higher in the Good group (*p* = 0.006) as shown in Fig. 3B. We therefore hypothesized that there was a relationship between the expression of ARHGAP10 and chemotherapy response, and the combination of epirubicin, cyclophosphamide and docetaxel may be less effective in breast cancer patients with low expression of ARHGAP10.

### ARHGAP10 is involved in cancer-related signaling pathways in breast cancer.

**Figure 4:**
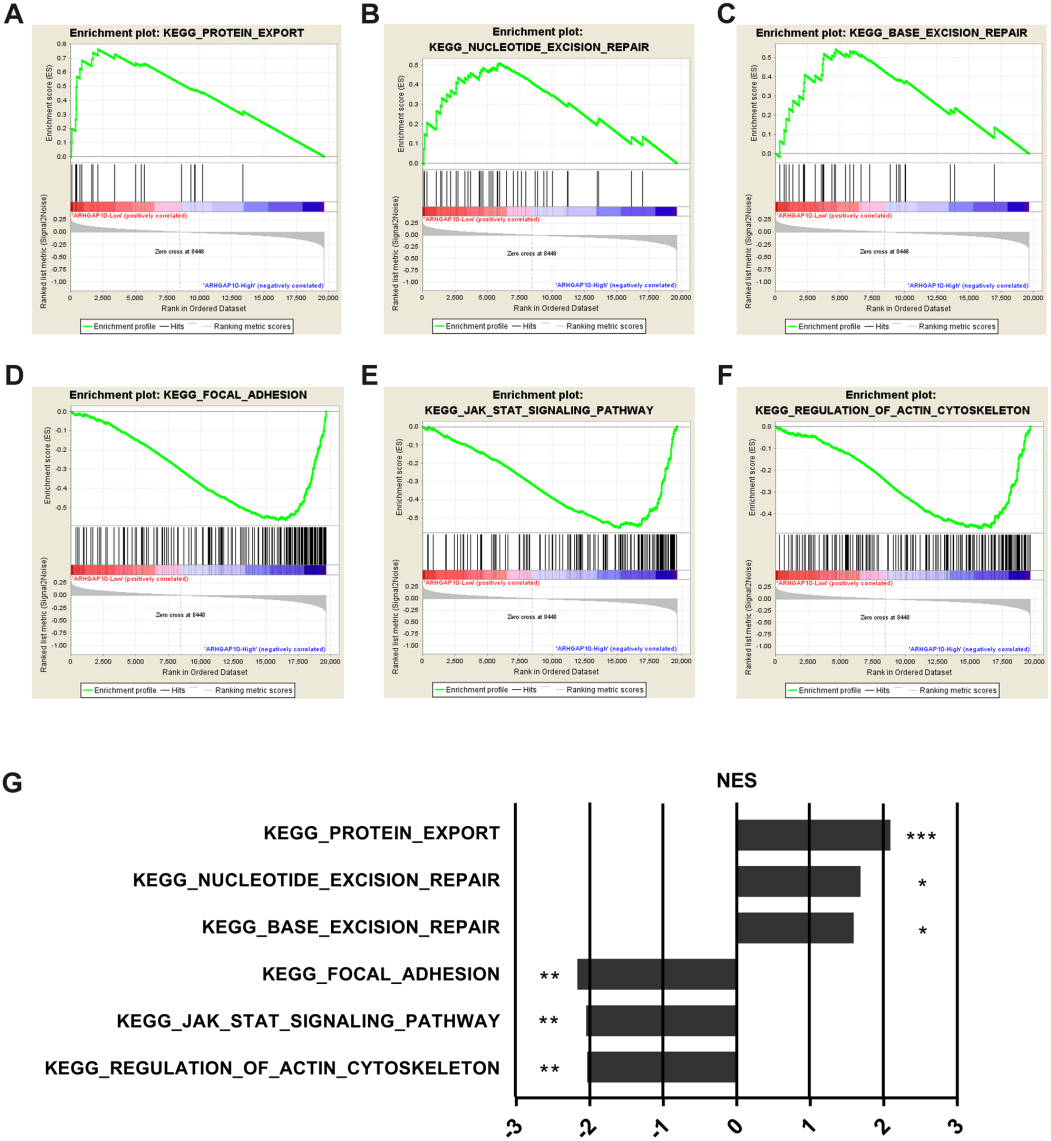
Gene Set Enrichment Analysis of ARHGAP10 in breast cancer. (A–C) Low expression of ARHGAP10 group was enriched in KEGG-protein export, KEGG-nucleotide excision repair and KEGG-base excision repair gene sets. (D–F) High expression of ARHGAP10 group was enriched in KEGG-focal adhesion, KEGG-JAK-STAT and KEGG-regulation of actin cytoskeleton gene sets. (G) The enriched signal pathways were summarized by the diagram. NES, normalized enrichment score. * *p* < 0.05, ** *p* < 0.01, *** *p* < 0.001.

To further examine the biological function and associated signal pathways that ARHGAP10 possibly involved in, GSEA was performed (Subramanian et al., 2005) using data downloaded from TCGA. A total of 1,097 breast cancer samples were divided into two groups by the median expression of ARHGAP10, namely ARHGAP10-low group and ARHGAP10-high group. The GSEA results indicated that protein export, nucleotide excision repair and base excision repair gene sets were markedly enriched in the ARHGAP10-low group (Figs. 4A–4C). Focal adhesion, JAK-STAT and actin cytoskeleton gene sets were evidently enriched in the ARHGAP10-high group (Figs. 4D–4F) with a nominal *p*-value <0.05 and FDR <0.25. The normalized enrichment scores of the signal sets were summarized in Fig. 4G.

## Discussion

Little is elucidated about ARHGAP10 in breast cancer before. Here, we showed that ARHGAP10 expression was downregulated at both the transcriptional and protein levels in breast cancer. Intriguingly, low expression of ARHGAP10 was more frequent in patients with cTNM stage II than in those with stage I. In addition, ARHGAP10 expression was lower in invasive ductal breast carcinoma than in adjacent normal tissues, whereas ARHGAP10 expression did not differ between ductal breast cancer in situ and normal tissues, as determined using the Radvanyi Breast dataset in Oncomine (Radvanyi et al., 2005). These results suggested that ARHGAP10 was more involved in tumor progression than initiation. ARHGAP10 was not only downregulated in breast cancer but also in other cancers including bladder cancer, esophageal cancer, liver cancer, prostate adenocarcinoma, rectum adenocarcinoma, skin cutaneous melanoma, uterine carcinosarcoma according to GEPIA. Luo, Teng and Li et al. have proved the reduced expression of ARHGAP10 is downregulated in ovarian cancer (Luo et al., 2016), lung cancer (Teng et al., 2017) and gastric cancer (Li et al., 2017). However, we found ARHGAP10 was upregulated in lymphoid neoplasm diffuse large B-cell lymphoma, acute myeloid leukemia and thymoma with the use of GEPIA. These results support the involvement of ARHGAP10 in various malignant tumors.

An increasing number of studies have identified massive genes with abnormal expression patterns in tumors when paralleled with normal tissues due to many mechanisms. To clarify the mechanisms underlying ARHGAP10 downregulation in breast cancer, we analyzed the region 2000bp upstream of ARHGAP10, and showed the presence of CpG islands in this region. Because methylations on CpG islands can lead to improper downregulation expression of genes (Illingworth & Bird, 2009), we speculated that this could be a potential underlying mechanism for the low expression of ARHGAP10. Regulation from miRNAs modulate the expression of genes (Bartel, 2004). Li et al. (2017) showed that miR-3174 targets ARHGAP10 and downregulates it expression in gastric cancer. Lu et al. (2012) predicted that ARHGAP10 may be a target of miR-214. However, miRNAs and other non-coding RNAs that modulate the expression of ARHGAP10 in breast cancer are unknown. Further investigations of the mechanisms underlying the downregulation of ARHGAP10 in breast cancer and other cancers are needed. Identifying the mechanisms or genes involved may shed new light on cancer initiation, progression, clinical diagnosis and treatment.

ARHGAP10, also known as GRAF2 or PS-GAP, converts the active GTP-bound state of GTPase into the inactive GDP-bound state. Rho GTPases such as Cdc42 and RhoA can interact with ARHGAP10 and can be inactivated by ARHGAP10 in vivo and in vitro (Shibata et al., 2001). Theses Rho GTPases could be the downstream targets of ARHGAP10 and mediate its biological functions. Luo et al. (2016) demonstrated the interaction between ARHGAP10 and Cdc42, and suggested that ARHGAP10 exerts its tumor suppressor function through Cdc42. Several studies have investigated these two Rho GTPases in tumors. Inhibition of RhoA can suppress the migration and invasion of triple negative breast cancer cells (Li et al., 2017b). Targeting Cdc42 by miRNAs or small molecules may retard the metastasis process in cancers (Xiao et al., 2018). A 15-year follow-up study of breast cancer showed that nuclear location of RhoA and Cdc42 correlates with less nodal metastasis, and high nuclear Cdc42 is related to low-grade tumor characteristics, whereas cytoplasmic localization of Cdc42 is associated with higher grade, higher Ki-67 index, and larger tumor size (Chrysanthou et al., 2017). In the present study, we found that ARHGAP10 was located at both nucleus and cytoplasm. Elucidating the mechanisms regulating the localizations and interactions between GTPases and GTPases-activating proteins may explain the distinct location-induced features of GTPases. Abnormally high expression of Cdc42 was detected in other types tumors such as cervical squamous cell carcinoma (Ma et al., 2013) and melanoma (Maldonado & Dharmawardhane, 2018). It is worth noting that the relationship between activity and expression of an enzyme is important, as high expression but low activity may act less than low expression but high activity, Pauline Croisé mentioned this issue about Rho GTPases in cancer (Croise et al., 2017). We therefore propose that the function of ARHGAP10 depends partly on the activity regulation of Rho GTPases such as RhoA and Cdc42.

We analyzed 190 breast cancer tissue samples and 30 adjacent normal tissues, and the results showed that ARHGAP10 was downregulated in breast cancer tissues and low expression was correlated with high Ki-67 index and advanced cTNM stage. The targets of ARHGAP10, i.e., GTPases, play a role in cytoskeleton formation and proliferation (Rittinger et al., 1997) and are involved in migration and invasion of breast cancer cells (Li et al., 2017b). This suggested that ARHGAP10 played a role in metastasis and proliferation in breast cancer. However, our data did not fully support this hypothesis, as ARHGAP10 was associated with proliferation but not metastasis. These results could be attributed to the small sample size and the lack of cell or animal experiments; however, it is also possible that ARHGAP10 had a different biological function. The exact biological role of ARHGAP10 needs to be examined in further studies.

The heterogeneity of breast cancer can lead to different patient outcomes (Song et al., 2016), and personalized medicine against specific molecules associated with heterogeneity improves the curative effect of anti-cancer treatments (Schnitt, 2010). Accordingly, we investigated whether ARHGAP10 was involved in the response to treatment. High throughput sequencing is used extensively in oncology to identify critical genes as biomarkers for early detection, diagnosis and outcome prediction (Kulasingam & Diamandis, 2008). We analyzed the public data GSE45898, which was established in a study investigating the response to chemotherapy in breast cancer. The results suggested that low expression of ARHGAP10 indicated a poor response to epirubicin, cyclophosphamide and docetaxel. Li et al. (2017) also found that low expression of ARHGAP10 can lead to cisplatin resistance in gastric cancer cell lines. These studies suggested that ARHGAP10 was involved in the regulation of drug resistance. Cdc42 can activate and increase Ras and EGFR signaling, and Cdc42 is a putative therapeutic target in breast, colon, lung cancer and pancreatic cancers (Aguilar, Zhou & Lu, 2017). In a recent study, Cdc42 was reported as a promising target for cancer treatment (Maldonado & Dharmawardhane, 2018). However, whether the role of ARHGAP10 in the response to chemotherapy is associated with Cdc42 remains unknown. ARHGAP10 could be a potential biomarker for predicting response to chemotherapy and may be involved in dysregulated drug responses.

GSEA analysis provided evidence that ARHGAP10 was involved in the protein export, nucleotide excision repair, base excision repair, focal adhesion, JAK-STAT and actin cytoskeleton signaling pathways. Base excision repair is enriched for ARHGAP10 in ovarian cancer (Luo et al., 2016). Metastasis and the Wnt signaling pathway are regulated by ARHGAP10 in lung cancer cells (Teng et al., 2017). Collectively, these findings suggested that ARHGAP10 was involved in different aspects of cancer. It would therefore be of value to investigate how ARHGAP10 is involved in these signaling pathways to play its biological roles in breast cancer.

## Conclusions

In summary, the expression of ARHGAP10 is downregulated in breast cancer, and relative low expression of ARHGAP10 is correlated with malignant characteristics such as a high Ki-67 index, advanced cTNM stage, poor response to chemotherapy and low RFS rates. GSEA results indicate that ARHGAP10 is involved in certain cancer-related signaling pathways. Additional studies are required to gain more insights into the role of ARHGAP10 in breast cancer.

##  Supplemental Information

10.7717/peerj.7431/supp-1 Figure S1 The original image of Fig. 2A magnified at 40X The original image of the staining of ARHGAP10 in Fig. 2A at the magnification of 40X.

10.7717/peerj.7431/supp-2 Figure S2 The original image of Fig. 2A magnified at 200X The original image of the staining of ARHGAP10 in Fig. 2A at the magnification of 200X.

10.7717/peerj.7431/supp-3 Figure S3 The original image of Fig. 2A magnified at 400X The original image of the staining of ARHGAP10 in Fig. 2A at the magnification of 400X.

10.7717/peerj.7431/supp-4 Figure S4 The original image of Fig. 2B magnified at 40X The original image of the staining of ARHGAP10 in Fig. 2B at the magnification of 40X.

10.7717/peerj.7431/supp-5 Figure S5 The original image of Fig. 2B magnified at 200X The original image of the staining of ARHGAP10 in Fig. 2B at the magnification of 200X.

10.7717/peerj.7431/supp-6 Figure S6 The original image of Fig. 2B magnified at 400X The original image of the staining of ARHGAP10 in Fig. 2B at the magnification of 400X.

10.7717/peerj.7431/supp-7 Figure S7 The original Western Blot for the band of ARHGAP10

10.7717/peerj.7431/supp-8 Figure S8 The original Western Blot for the band of GAPDH

10.7717/peerj.7431/supp-9 Figure S9 The original images of the staining of ARHGAP10 in Fig. 2 (middle, above) at the magnification of 40x

10.7717/peerj.7431/supp-10 Figure S10 The original images of the staining of ARHGAP10 in Fig. 2A (middle, below) at the magnification of 40x

10.7717/peerj.7431/supp-11 Figure S11 The original images of the staining of ARHGAP10 in Fig. 2A (right, above) at the magnification of 40x

10.7717/peerj.7431/supp-12 Figure S12 The original images of the staining of ARHGAP10 in Fig. 2 (right, below) at the magnification of 40x

10.7717/peerj.7431/supp-13 Figure S13 The original images of the staining of ARHGAP10 in Fig. 2B (left) at the magnification of 40x

10.7717/peerj.7431/supp-14 Figure S14 The original images of the staining of ARHGAP10 in Fig. 2B (right) at the magnification of 40x

10.7717/peerj.7431/supp-15 Figure S15 Staining of epithelial marker and Ki-67 of Fig. 2A The bulk of tumor sample underwent serial sections for staining of various parameters, so the images of different parameters could hardly coincide. These parameters were previously stained and saved by Department of Pathology for the diagnosis of breast cancer.

10.7717/peerj.7431/supp-16 Figure S16 Staining of epithelial marker and Ki-67 of Fig. 2B The bulk of tumor sample underwent serial sections for staining of various parameters, so the images of different parameters could hardly coincide. These parameters were previously stained and saved by Department of Pathology for the diagnosis of breast cancer.

10.7717/peerj.7431/supp-17 Figure S17 Staining of ARHGAP10 and IgG

10.7717/peerj.7431/supp-18 Figure S18 The expression of ARHGAP10 in 13 pairs of primary breast cancer tissues and corresponding normal adjacent tissues detected by RT-qPCR assay and examined by paired *t*-test, *p* = 0.0225

10.7717/peerj.7431/supp-19 Figure S19 The expression of ARHGAP10 in lymphoid neoplasm diffuse large B-cell lymphoma (DLBC), acute myeloid leukemia (LAML) and thymoma (THYM)

10.7717/peerj.7431/supp-20 Figure S20 This relationships between Rho GTPases and ARHGAP10 were examined by Spearman correlation

10.7717/peerj.7431/supp-21 Figure S21 The expression of ARHGAP10 in ductal breast cancer in situ and normal tissues according to Radvanyi in Oncomine

10.7717/peerj.7431/supp-22 Table S1 The detail of the signal pathways enriched of ARHGAP10 including NES and nominal *p*-value

10.7717/peerj.7431/supp-23 Table S2 The raw data of RT-qRCR in 30 pairs of breast cancer tissues and corresponding adjacent normal tissues

10.7717/peerj.7431/supp-24 Table S3 Raw data of RT-qPCR of ARHGAP10 in breast cell lines

10.7717/peerj.7431/supp-25 Table S4 Raw data of the RT-qPCR of ARHGAP10 with the new primer in 13 pairs of breast cancer tissues and corresponding normal adjacent tissues

10.7717/peerj.7431/supp-26 Table S5 Correlation between the Ki-67 (cut-off value of 14%) and the expression of ARHGAP10 in 190 breast cancer cases

## Additional Information and Declarations

### Data Availability

The following information was supplied regarding data availability: The raw data are available as Supplemental Files.
