# Supplementary figures and images for: Downregulated expression of ARHGAP10 correlates with advanced stage and high Ki-67 index in breast cancer
Source: PeerJ. 2019 Aug 1;7:e7431. doi: 10.7717/peerj.7431 (PMC6679923; doi:10.7717/peerj.7431)

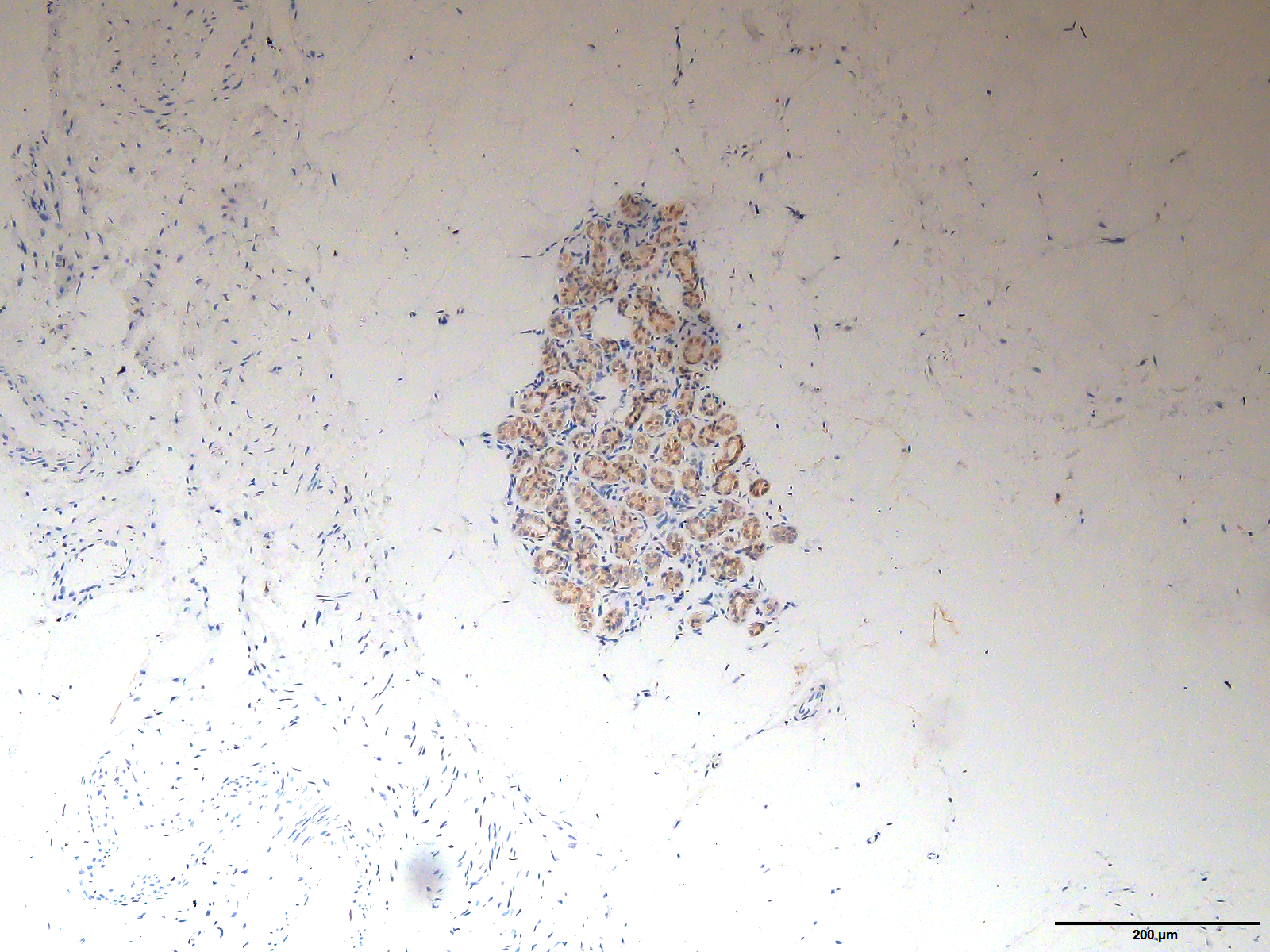

Supplement: Figure S1 — The original image of the staining of ARHGAP10 in Fig. 2A at the magnification of 40X. [file peerj-07-7431-s001.png]

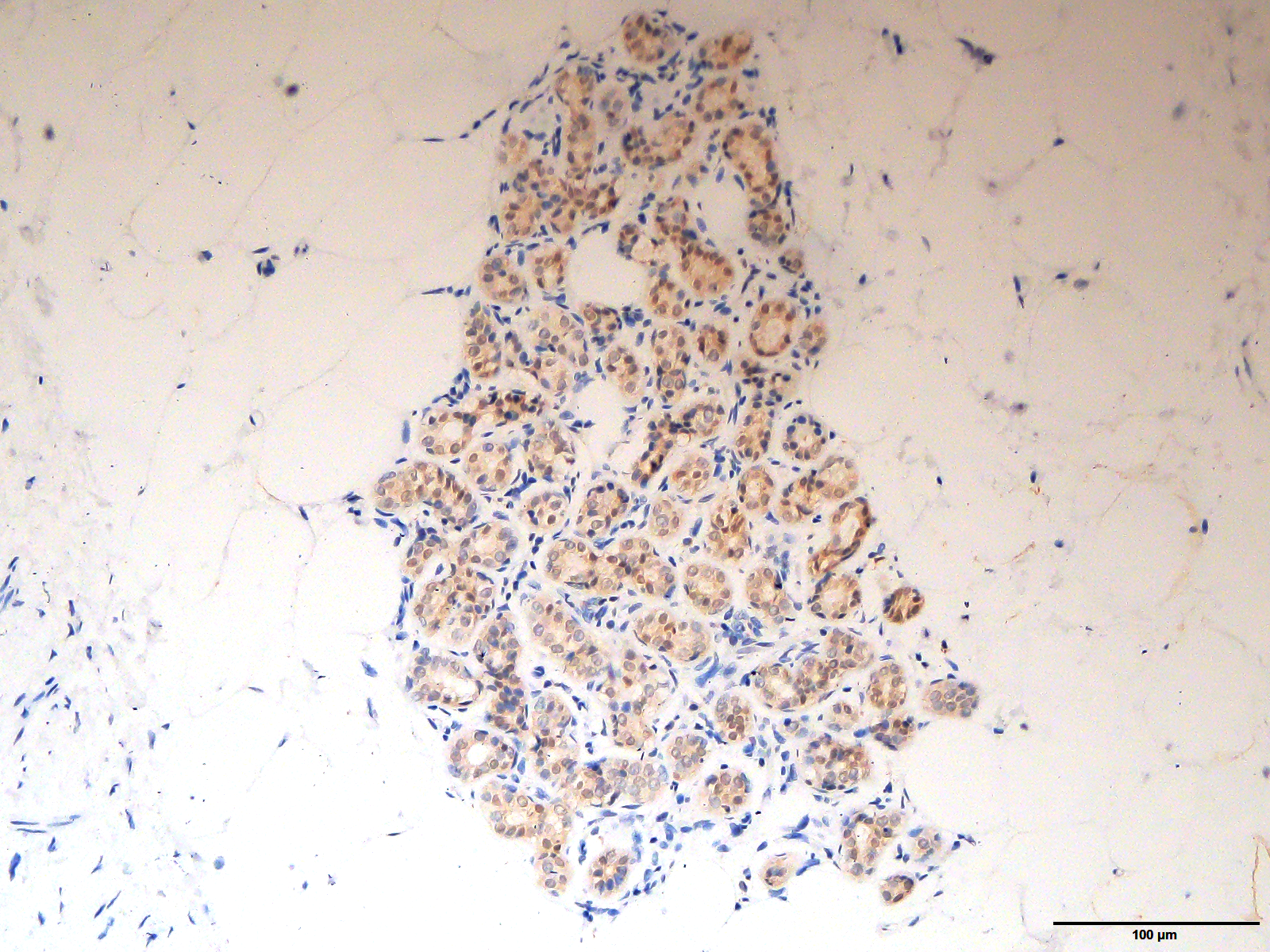

Supplement: Figure S2 — The original image of the staining of ARHGAP10 in Fig. 2A at the magnification of 200X. [file peerj-07-7431-s002.png]

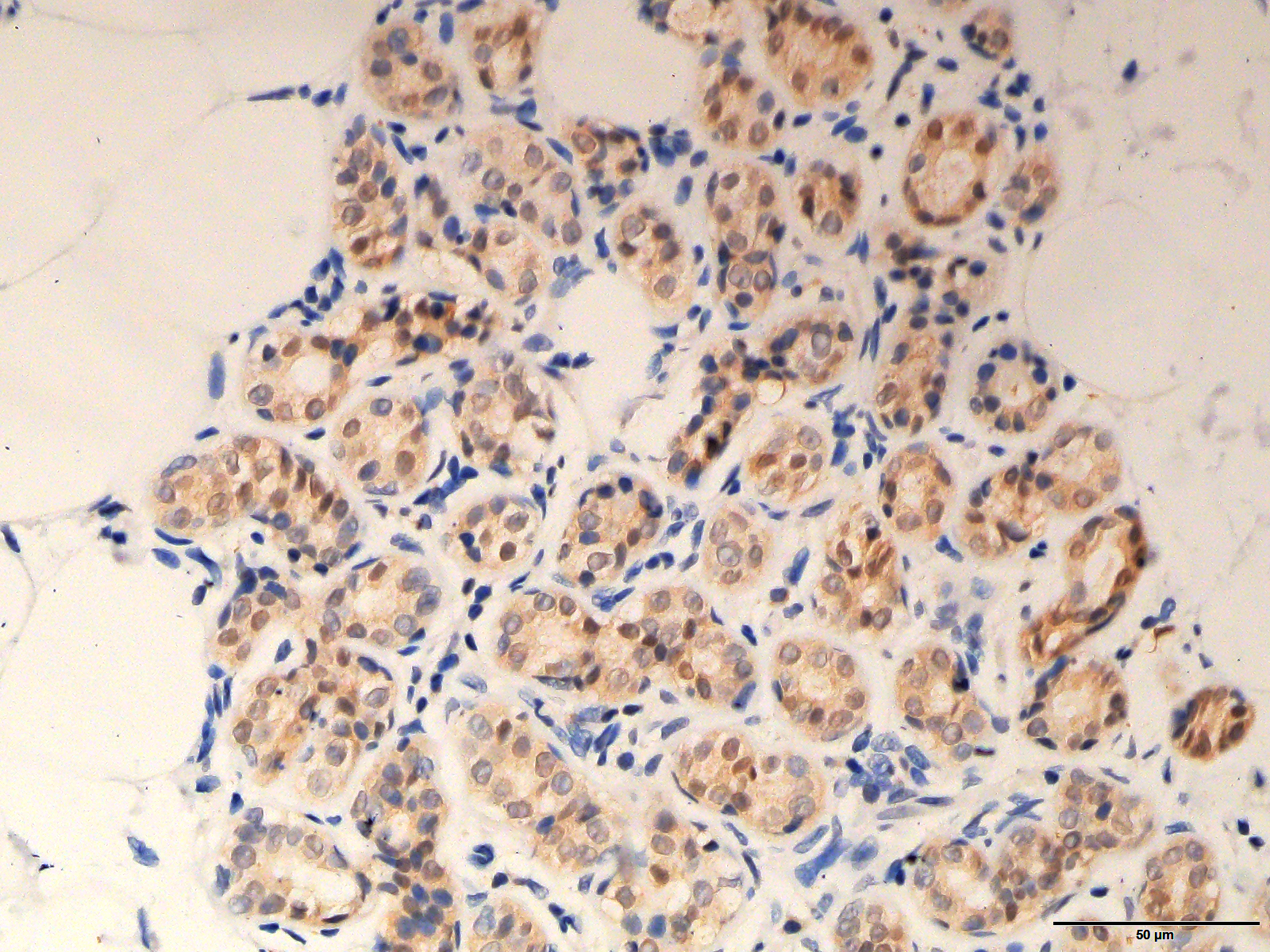

Supplement: Figure S3 — The original image of the staining of ARHGAP10 in Fig. 2A at the magnification of 400X. [file peerj-07-7431-s003.png]

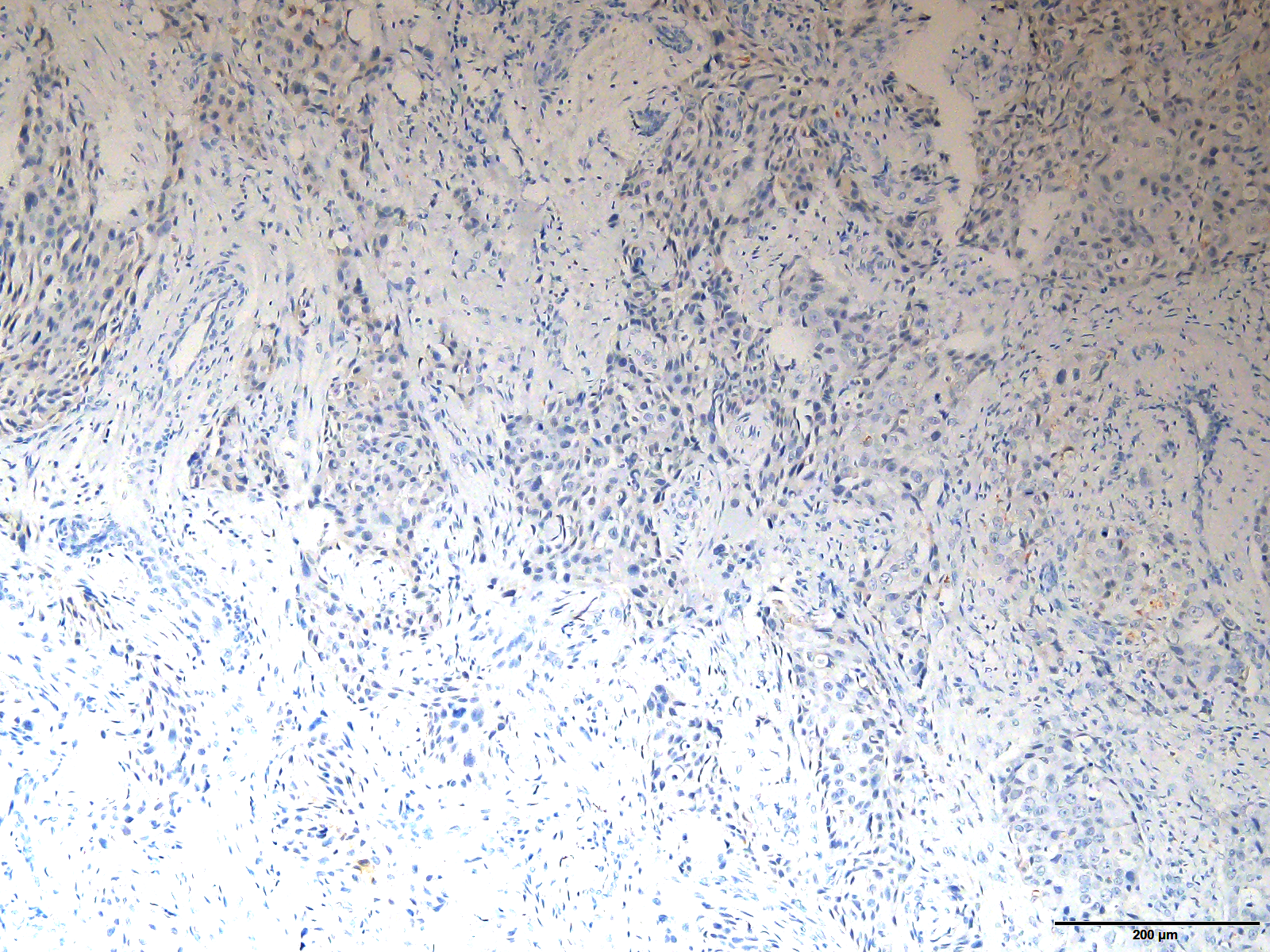

Supplement: Figure S4 — The original image of the staining of ARHGAP10 in Fig. 2B at the magnification of 40X. [file peerj-07-7431-s004.png]

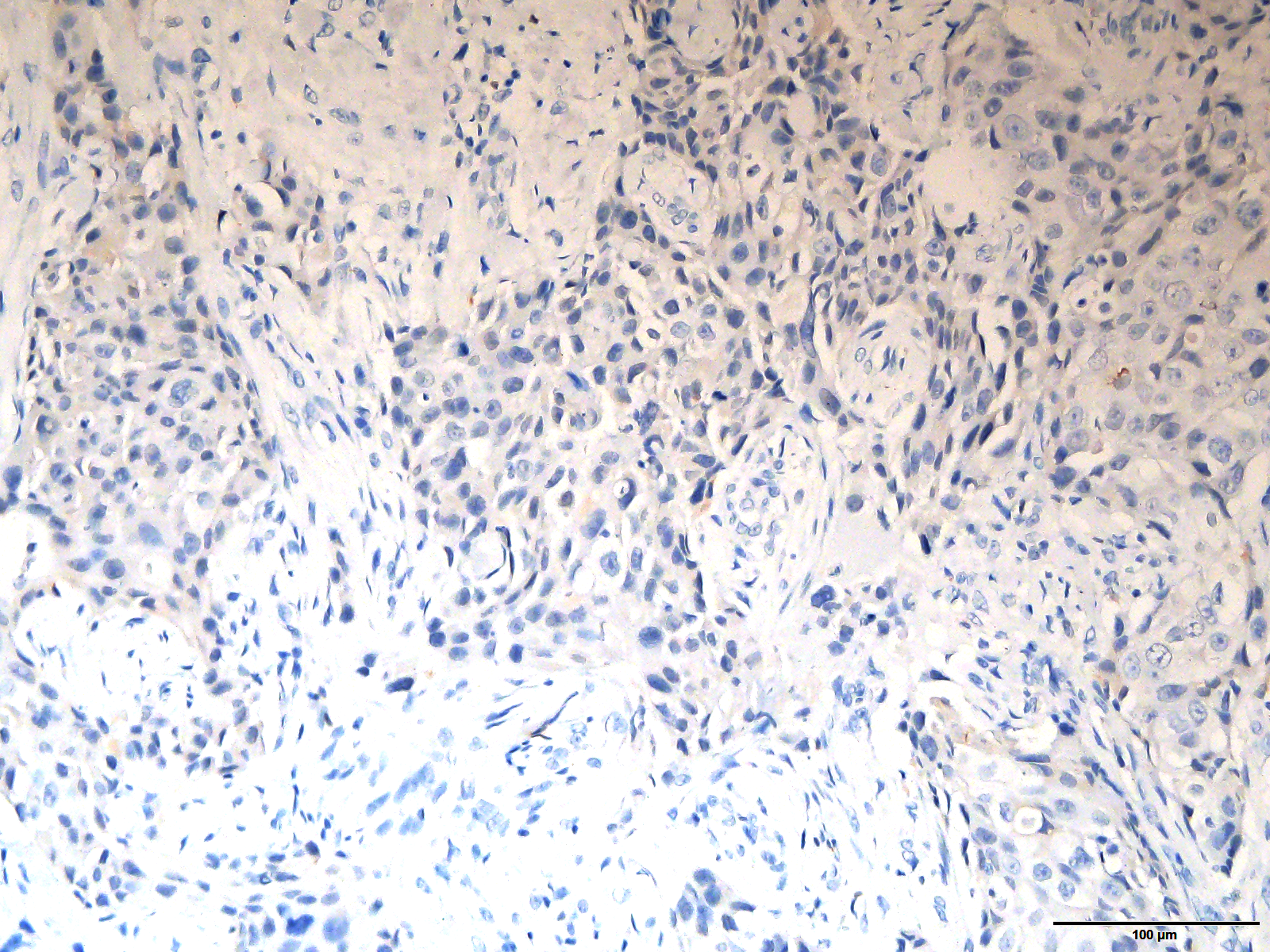

Supplement: Figure S5 — The original image of the staining of ARHGAP10 in Fig. 2B at the magnification of 200X. [file peerj-07-7431-s005.png]

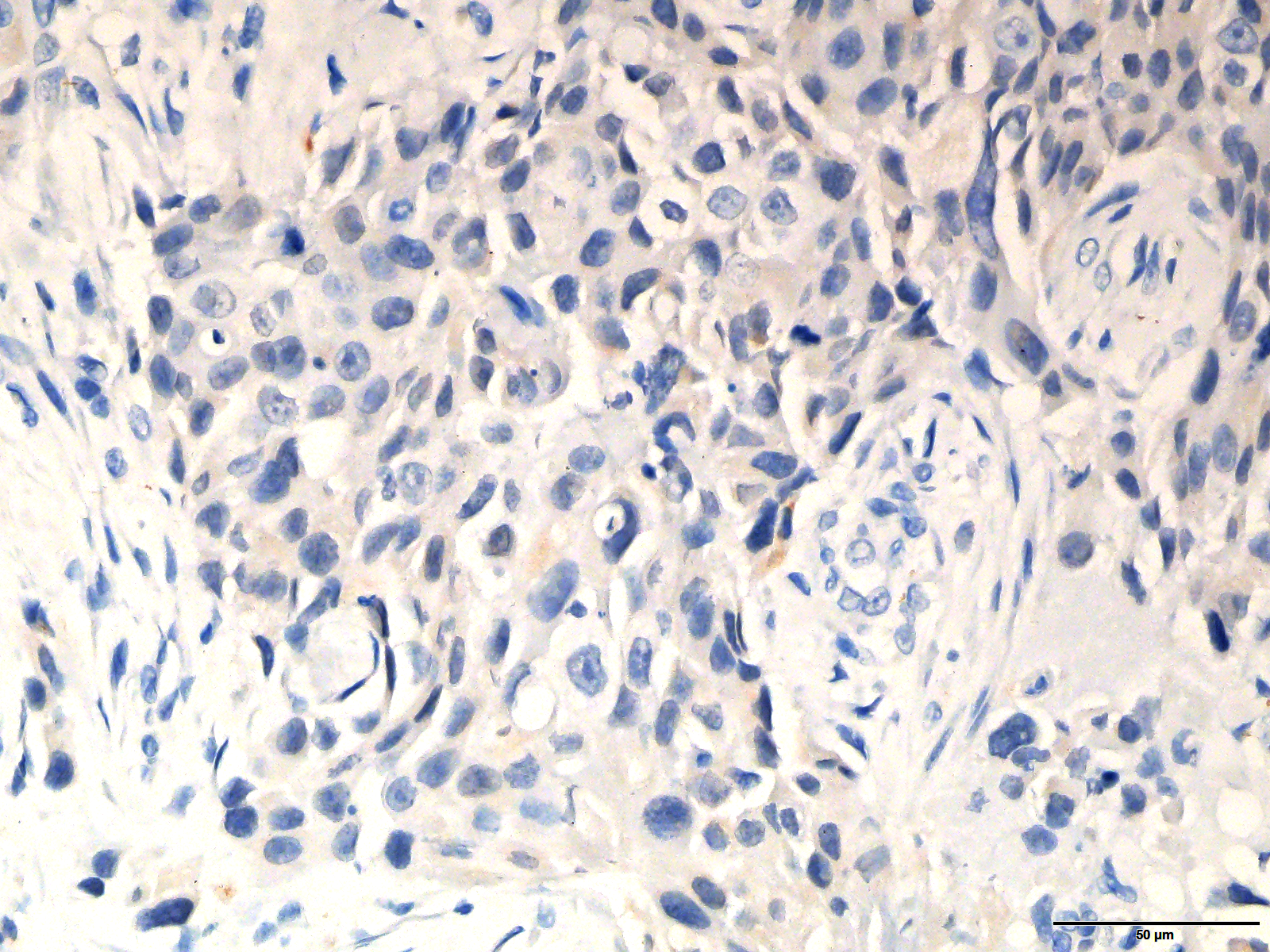

Supplement: Figure S6 — The original image of the staining of ARHGAP10 in Fig. 2B at the magnification of 400X. [file peerj-07-7431-s006.png]

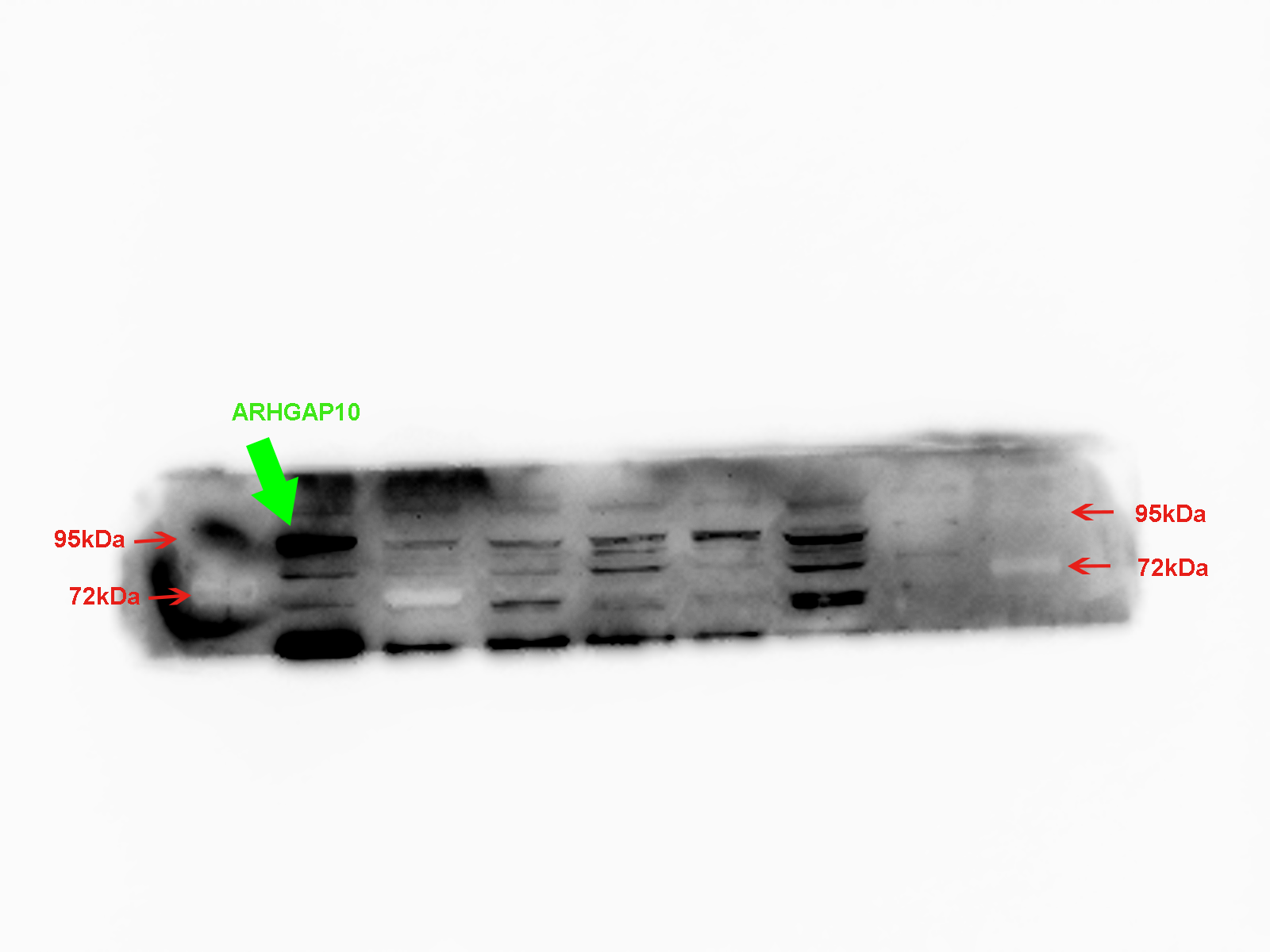

Supplement: Figure S7 [file peerj-07-7431-s007.png]

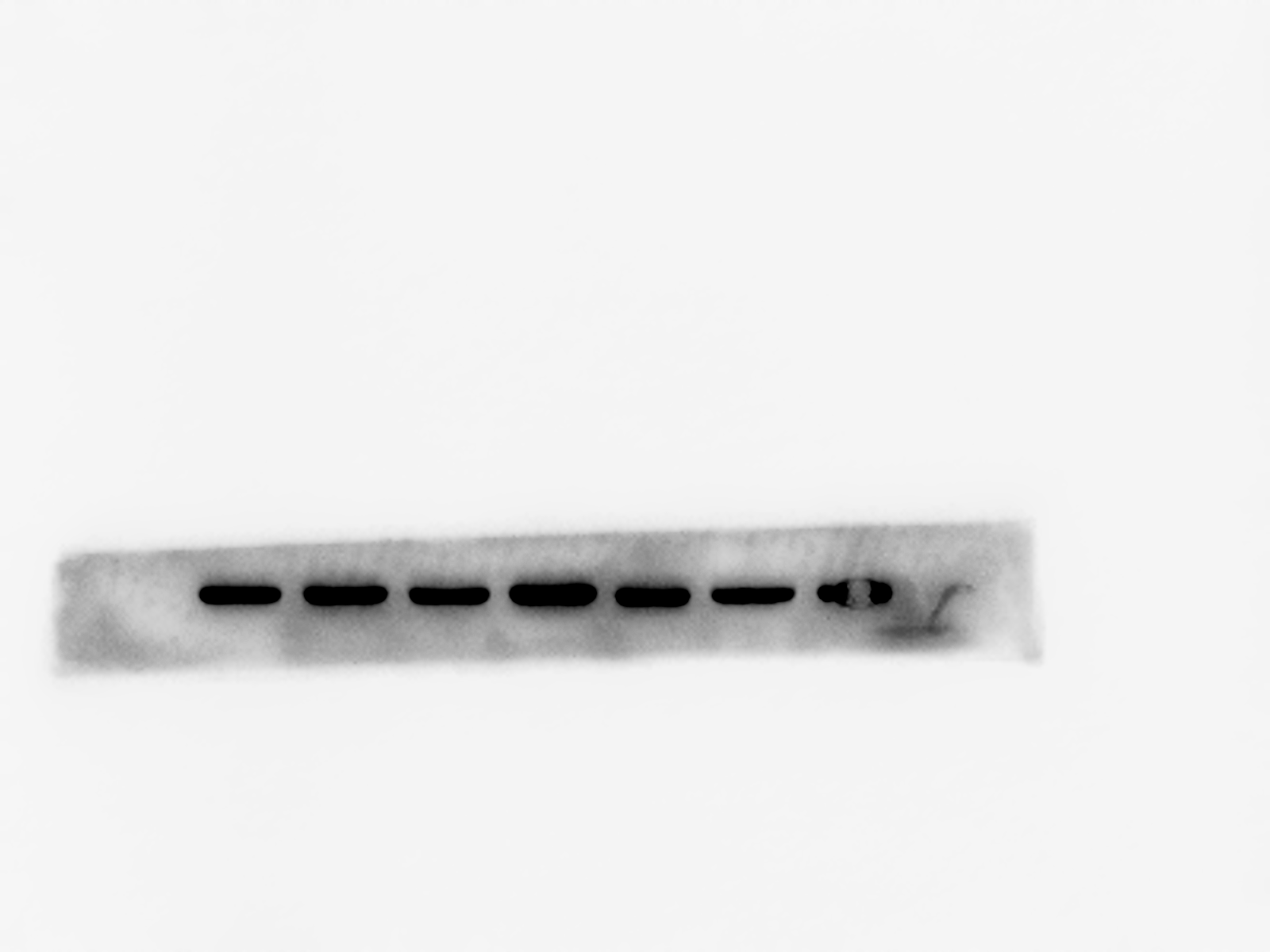

Supplement: Figure S8 [file peerj-07-7431-s008.png]

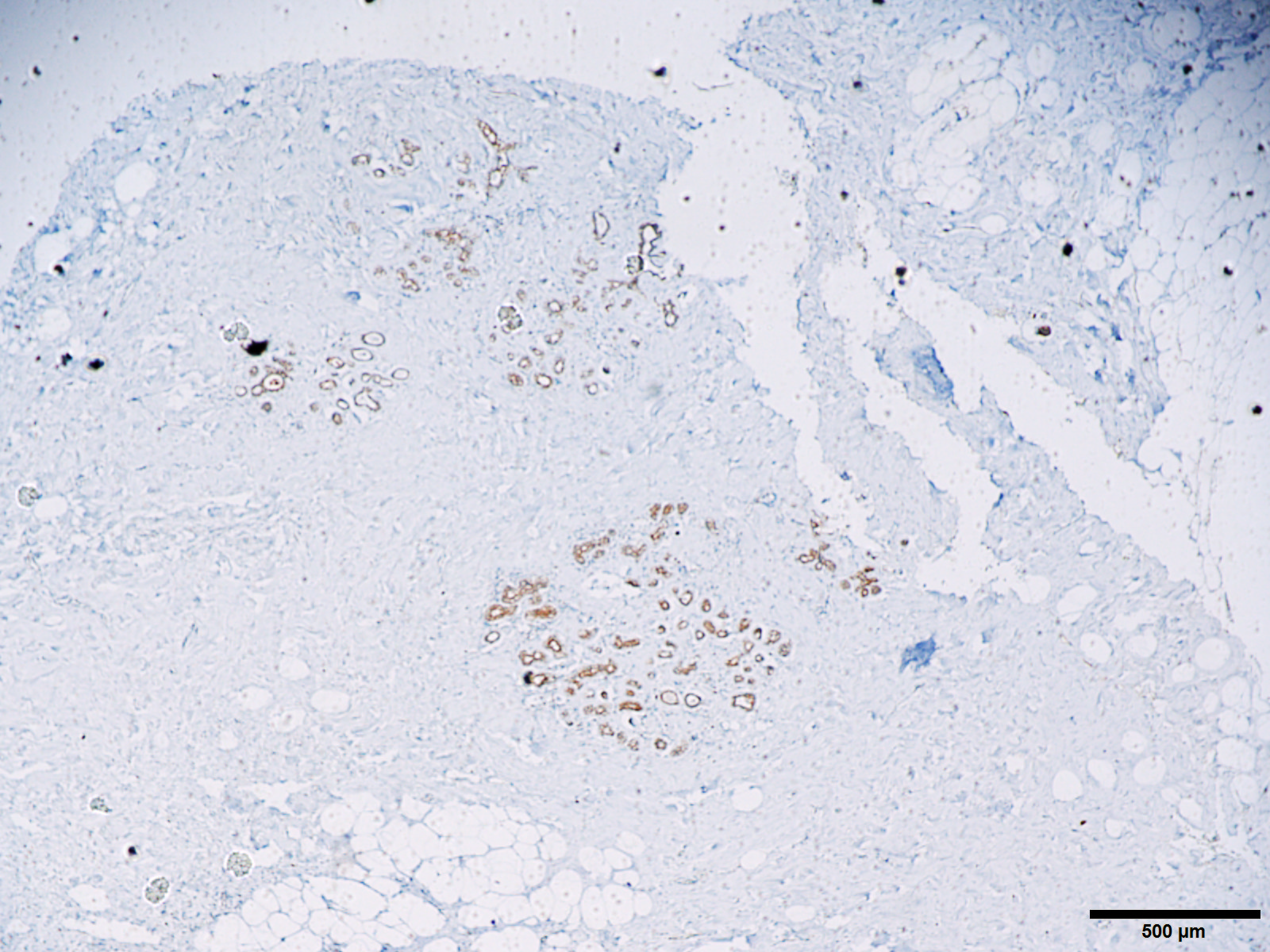

Supplement: Figure S9 [file peerj-07-7431-s009.png]

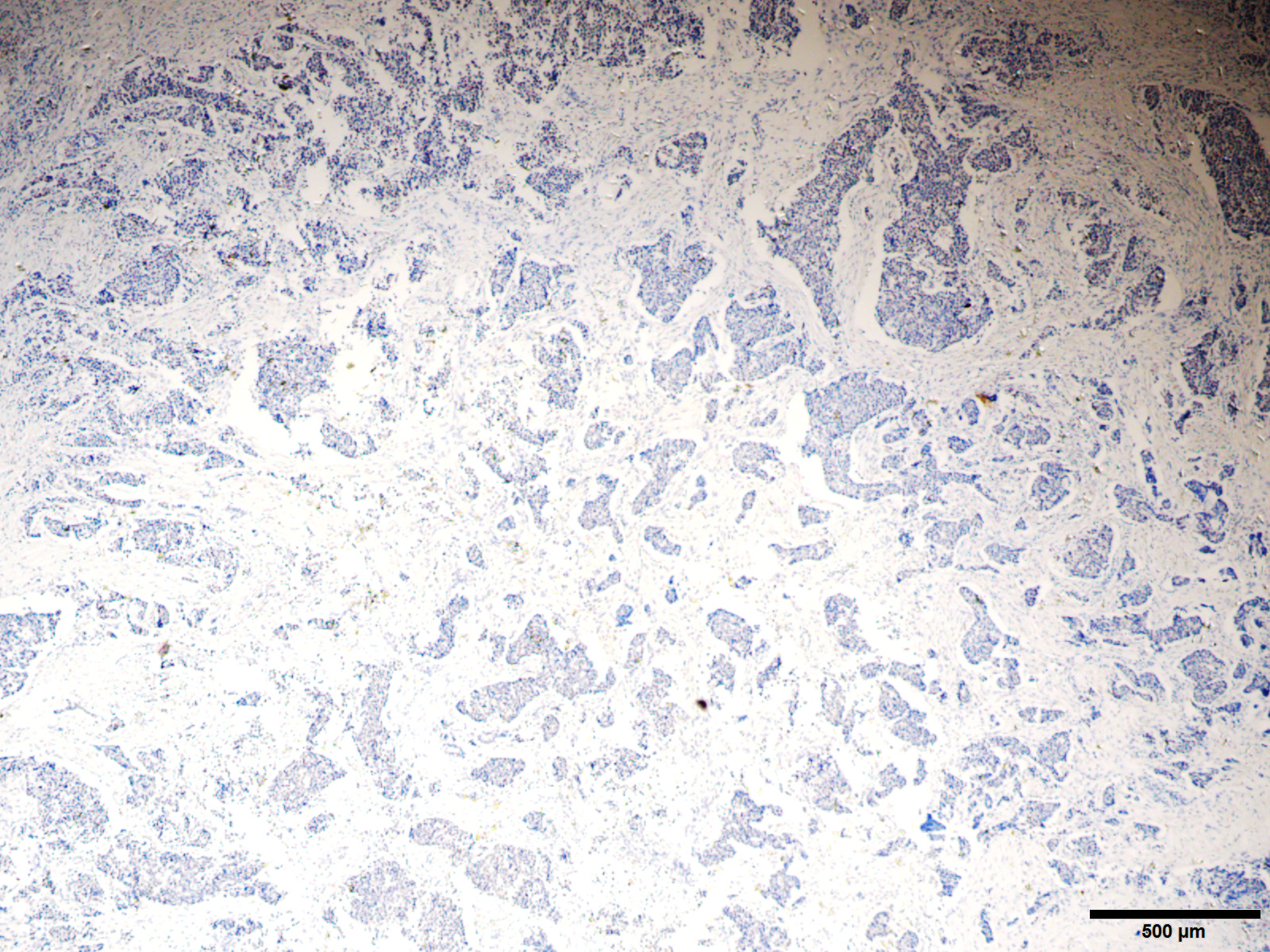

Supplement: Figure S10 [file peerj-07-7431-s010.png]

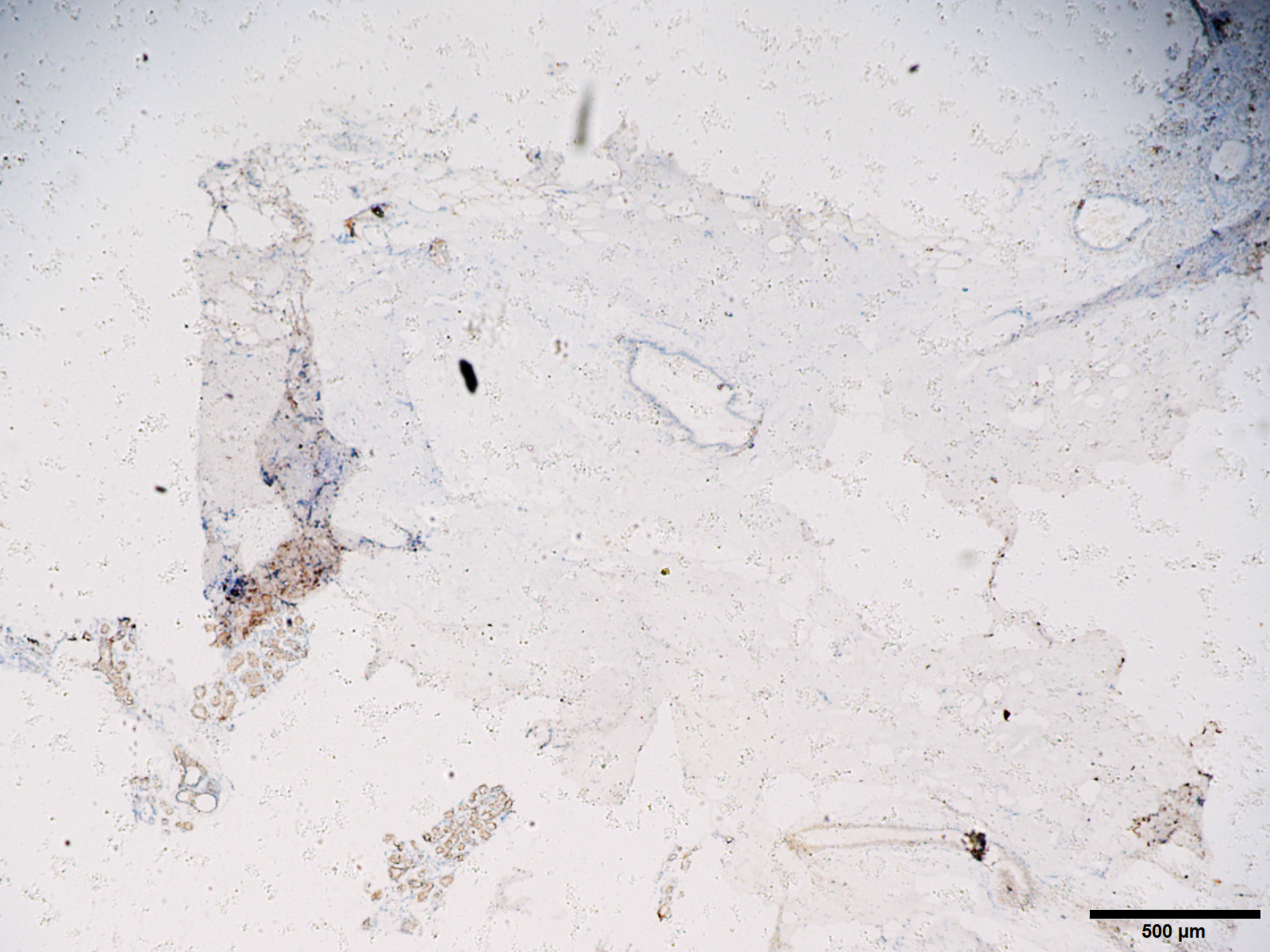

Supplement: Figure S11 [file peerj-07-7431-s011.png]

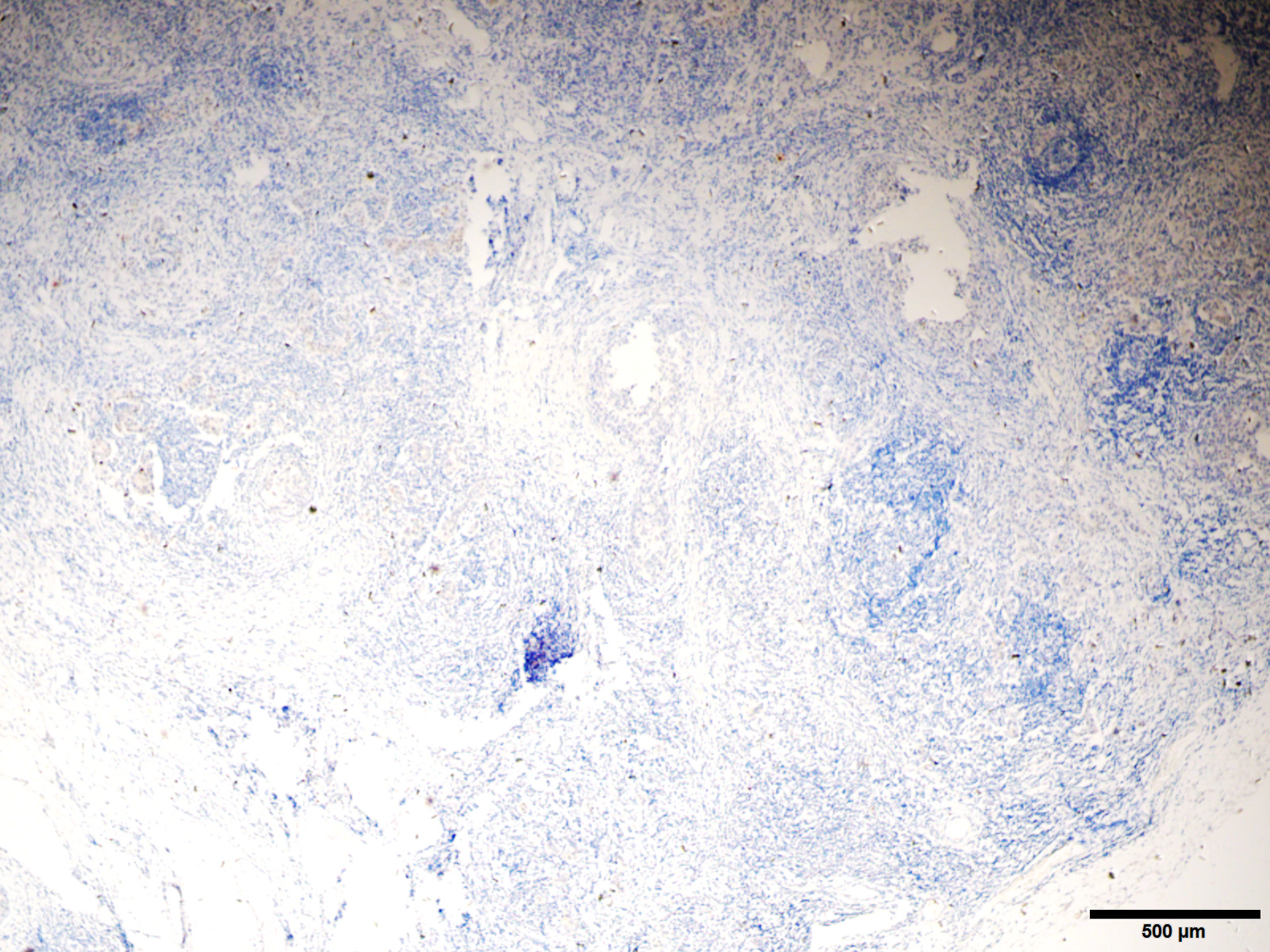

Supplement: Figure S12 [file peerj-07-7431-s012.png]

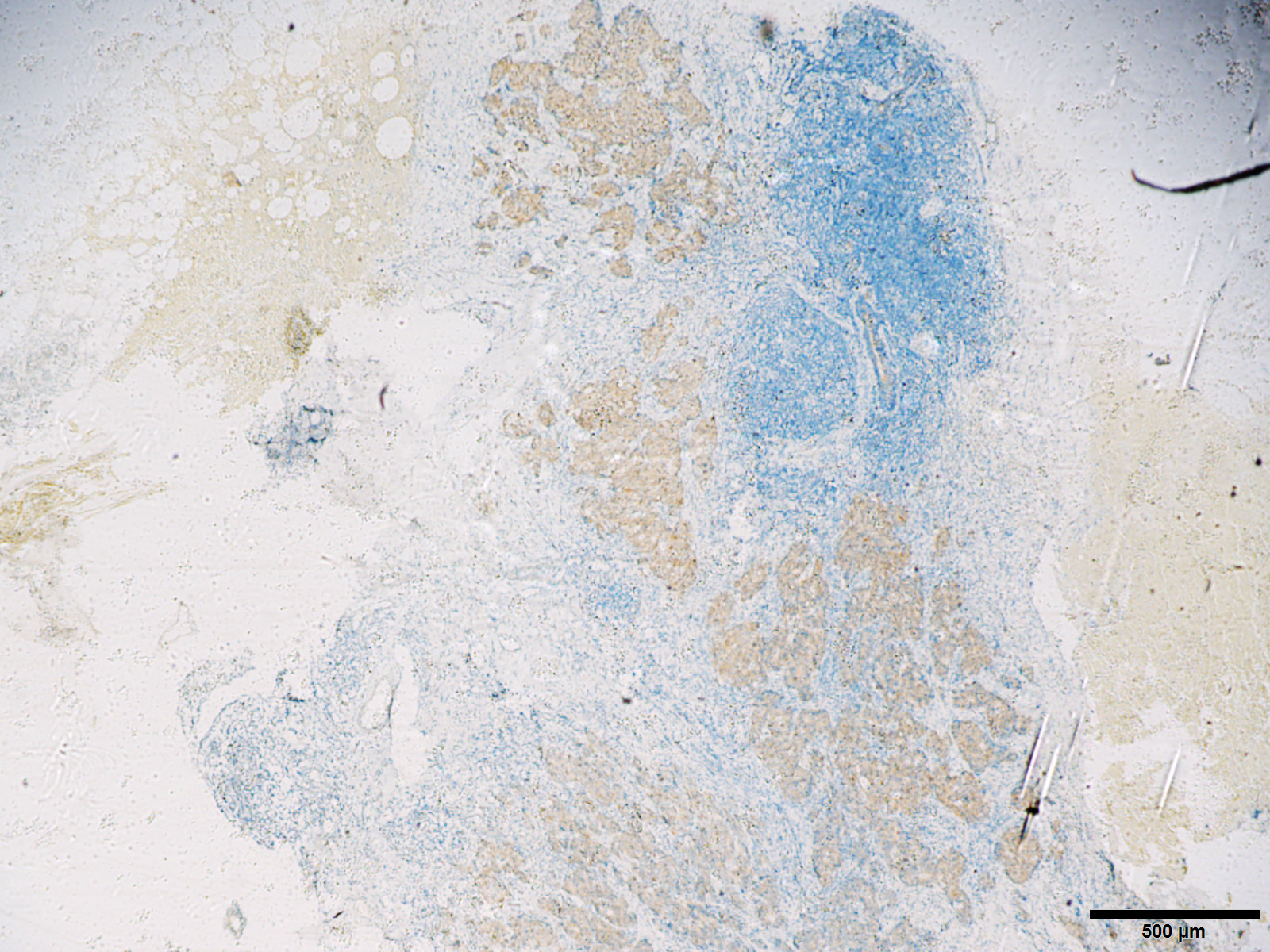

Supplement: Figure S13 [file peerj-07-7431-s013.png]

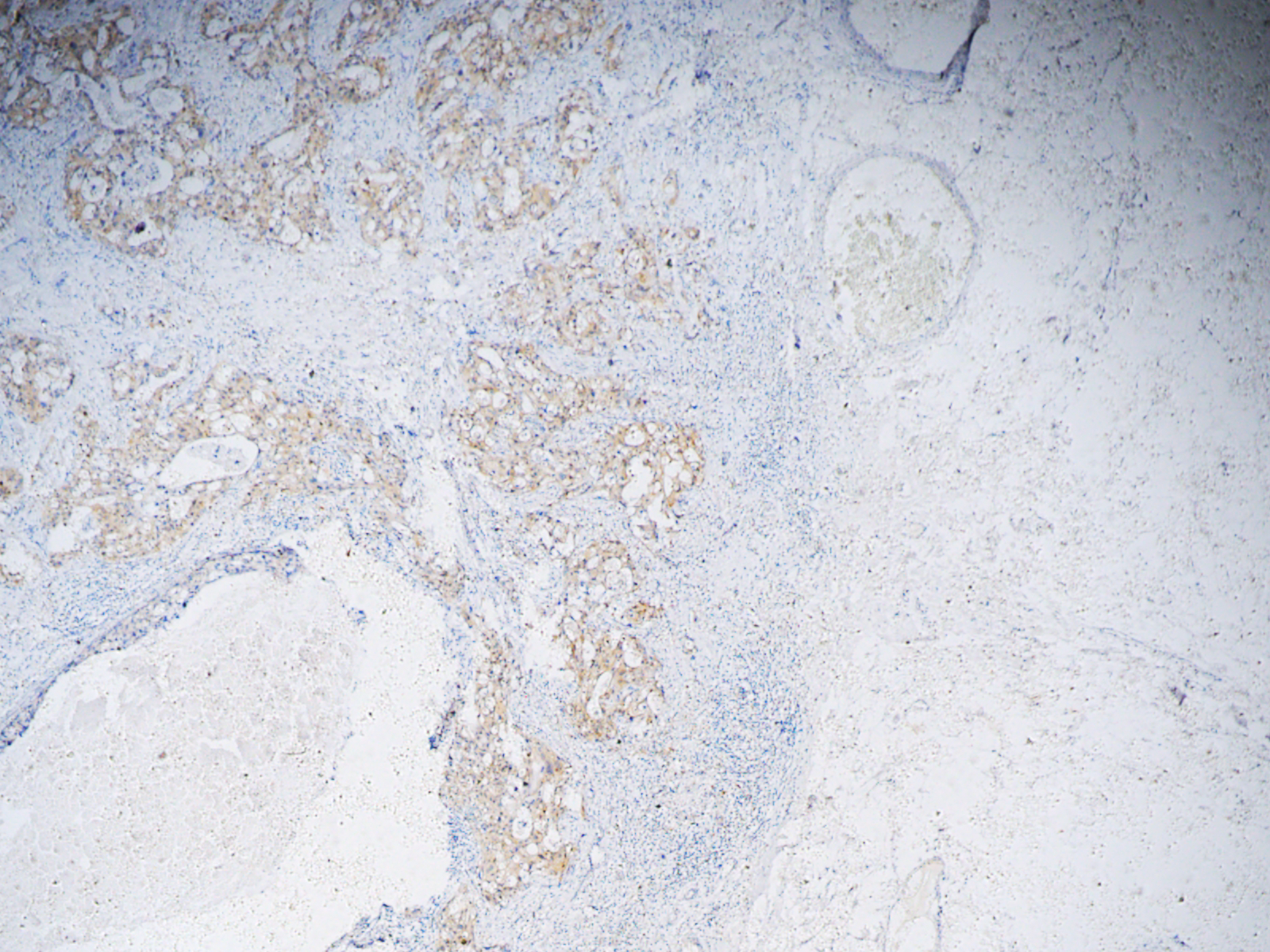

Supplement: Figure S14 [file peerj-07-7431-s014.png]

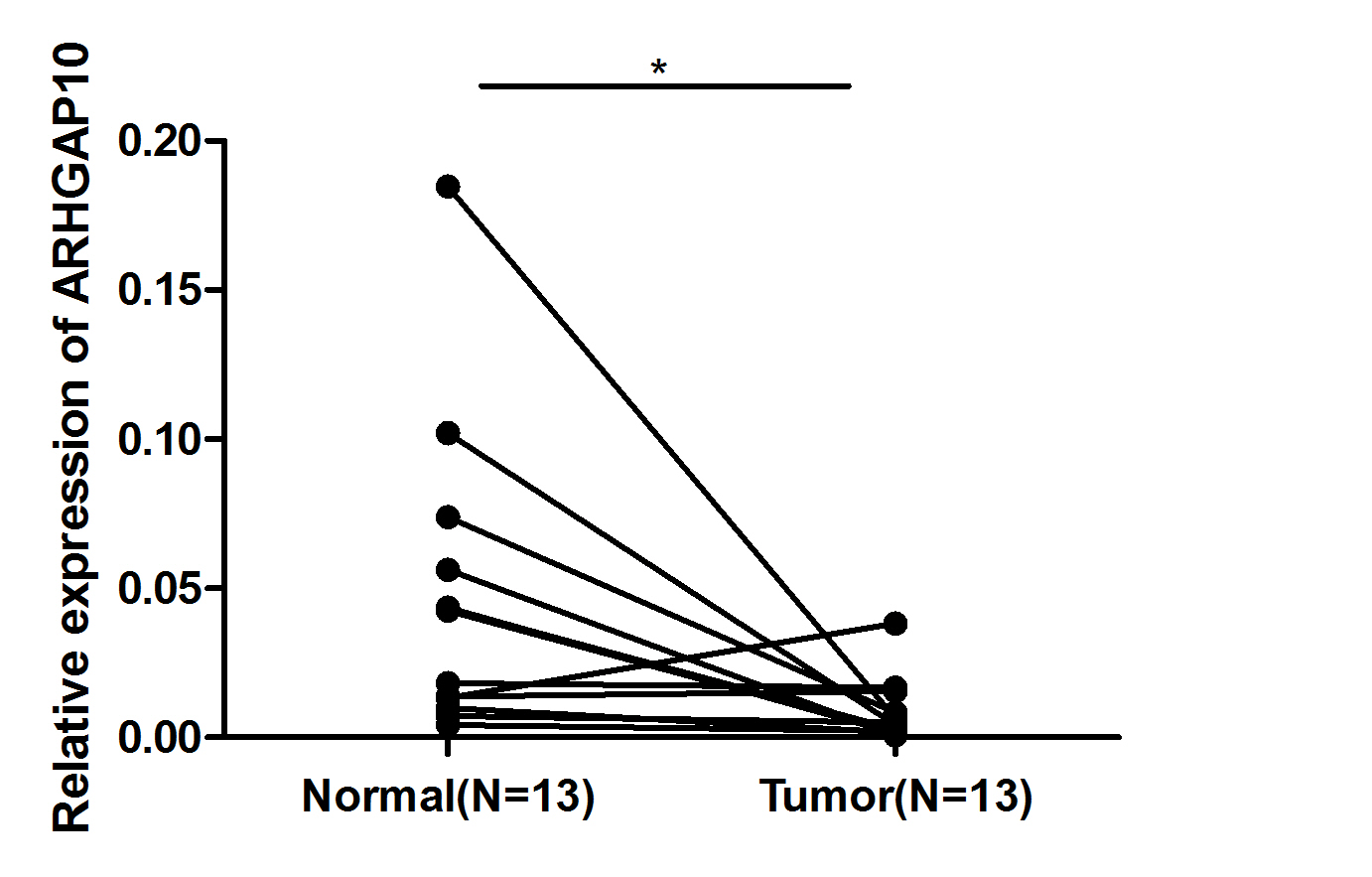

Supplement: Figure S18 [file peerj-07-7431-s018.jpg]

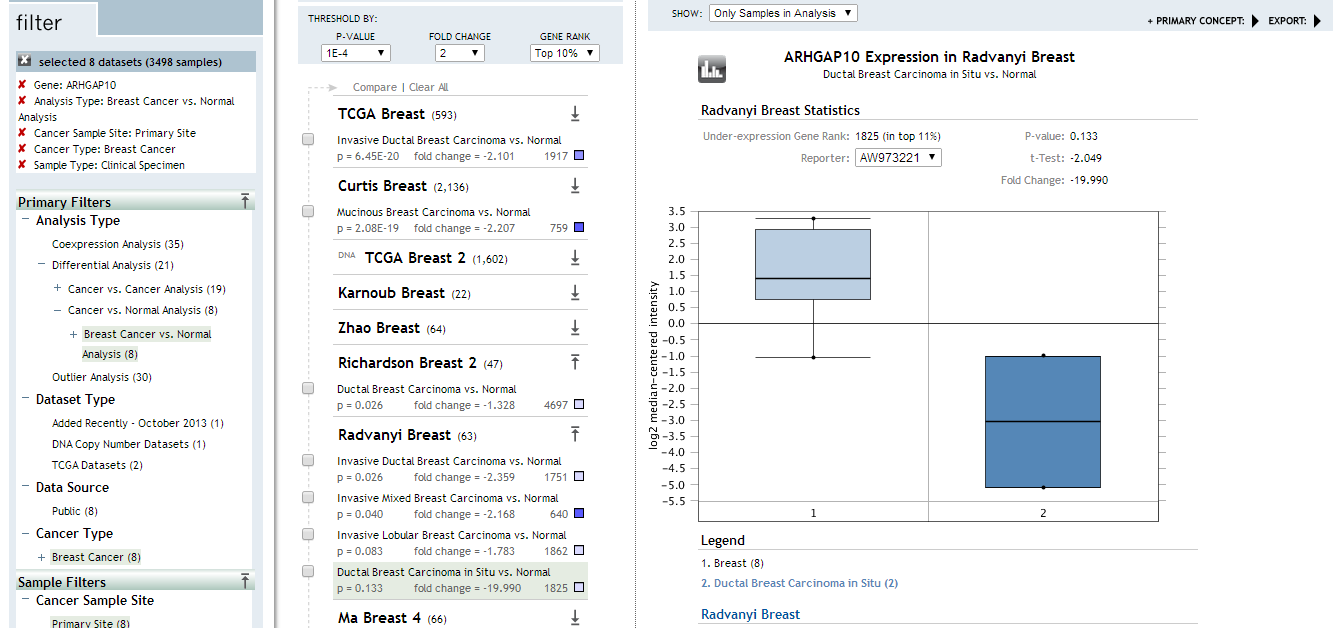

Supplement: Figure S21 [file peerj-07-7431-s021.png]
