# Supplementary material for: Downregulated expression of ARHGAP10 correlates with advanced stage and high Ki-67 index in breast cancer
Source: PeerJ. 2019 Aug 1;7:e7431. doi: 10.7717/peerj.7431 (PMC6679923; doi:10.7717/peerj.7431)
Supplement: Table S5 [file peerj-07-7431-s026.docx]

**Supplementary Table 5**

Correlation between the Ki-67 (cut-off value of 14%) and the expression of ARHGAP10 in 190 breast cancer cases.

| **Clinicopathological**  **Characteristics** | **Number** | **ARHGAP10 Expression** | | ***p* Value** |
| --- | --- | --- | --- | --- |
|  | (n=190) | Low | High |  |
| **Ki-67** |  |  |  | 0.032 |
| ≤14% | 55 | 33 | 22 |  |
| >14% | 135 | 102 | 33 |  |
